# Supplementary material for: Room-temperature high-precision printing of flexible wireless electronics based on MXene inks
Source: Nat Commun. 2022 Jun 9;13:3223. doi: 10.1038/s41467-022-30648-2 (PMC9184614; doi:10.1038/s41467-022-30648-2)
Supplement: Supplementary file 1 — Supplementary Information [file 41467_2022_30648_MOESM1_ESM.pdf]

Supplementary Information for

Room-temperature high-precision printing of flexible wireless electronics based on MXene inks

Yuzhou Shao[1], Lusong Wei[2], Xinyue Wu[1], Chengmei Jiang[1], Yao Yao[1], Bo Peng[1], Han Chen[1], Jiangtao Huangfu[2], Yibin Ying[1]*, Chuanfang (John) Zhang[3,4]* & Jianfeng Ping[1]*

[1] Laboratory of Agricultural Information Intelligent, School of Biosystems Engineering and Food Science, Zhejiang University, Hangzhou 310058, China

[2] Laboratory of Applied Research on Electromagnetics, Zhejiang University, Hangzhou 310027, China

[3] College of Materials Science & Engineering, Sichuan University, Chengdu, 610065, Sichuan, China

[4] Swiss Federal Laboratories for Materials Science and Technology (Empa), ETH Domain, Überlandstrasse 129, CH-8600 Dübendorf, Switzerland

Corresponding author: Yibin Ying, Chuanfang (John) Zhang, Jianfeng Ping

E-mail: ybying@zju.edu.cn; chuanfang.zhang@empa.ch; jfping@zju.edu.cn

Table of Contents

Contents	Page
Supplementary Figures …………………………………………………	3
• Supplementary Figures 1−33	
Supplementary Tables …………………………………………………	39
• Supplementary Tables 1−6	
Supplementary Notes …………………………………………………	47
• Material preparation and experimental methods	
Supplementary References …………………………………………………	59

Supplementary Figures

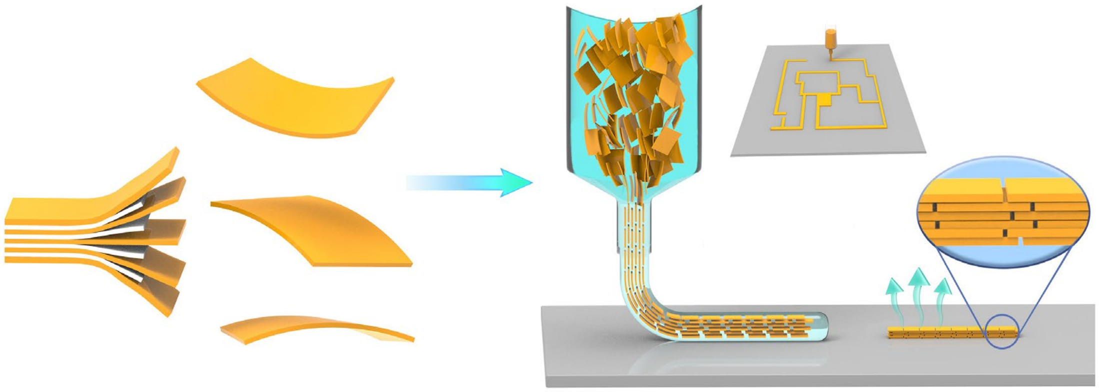

Supplementary Fig. 1 | Schematic diagram showing the preparation and extrusion printing of $Ti_3C_2T_x$ MXene ink. MXenes are a new 2D family of transition metal carbides and nitrides derived from the etched MAX precursor, with a general formula of $M_{n+1}X_nT_x$ (n = 1–4), where M stands for the transition metal, X denotes carbon or nitrogen, and T is the surface termination groups (e.g., -O, -OH, or -F). MXene aqueous inks have a large concentration range, allowing the rheological properties to be adjusted according to the printing method. Here, concentrated $Ti_3C_2T_x$ MXene aqueous inks were prepared *via* the modified MILD synthesis route, followed by optimized centrifugation and sonication, such that a high percent of single-layer flakes (>90%) with a narrow sheet diameter distribution are enabled. Consequently, the rheological properties of the as-formulated MXene inks fulfill the requirements of the extrusion printing very well, leading to high printing resolution and precision. Due to the shear force created in the nozzle of the printing head, $Ti_3C_2T_x$ nanosheets align themselves in the axial direction of the print head, forming highly-ordered liquid crystals within the extruded filaments and resulting in MXene films with densely stacked flakes in parallel to the substrate. Consequently, a high metallic electrical conductivity is enabled in the extrusion-printed MXene tracks. This result was also confirmed in subsequent experiments on the printed MXene films, such as XRD, SEM, and electrical measurements.

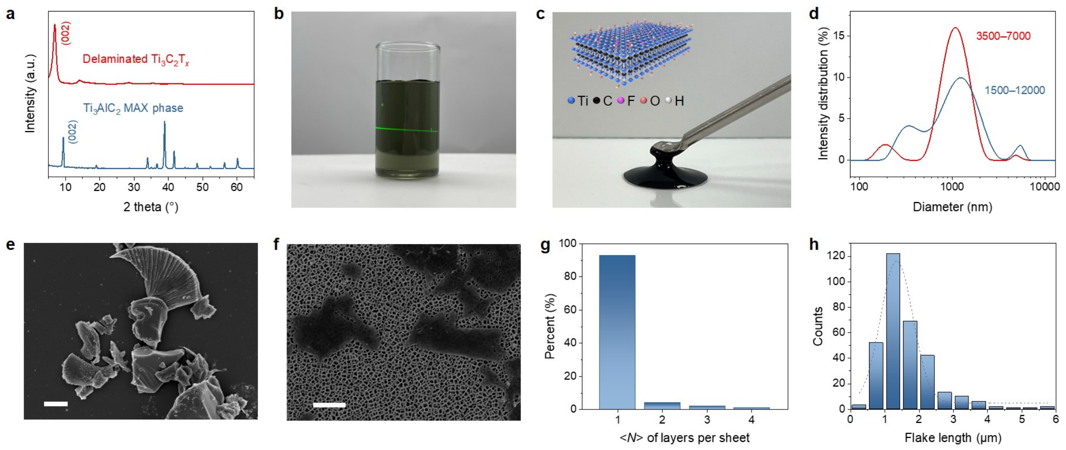

Supplementary Fig.2 | Characterizations of additive-free MXene aqueous inks. a, X-ray diffraction (XRD) patterns of Ti_3AlC_2 MAX phase and $Ti_3C_2T_x$ MXene flakes. The characteristic peaks near 39° of Ti_3AlC_2 completely disappeared after etching, indicating the removal of Al layer. Compared with the pristine Ti_3AlC_2 MAX, the (002) peak of $Ti_3C_2T_x$ MXene became broadened and shifted to a smaller angle, indicating the presence of fewer ordering crystals with increased d-spacing. These XRD results demonstrate that $Ti_3C_2T_x$ nanosheets were successfully prepared from the Ti_3AlC_2 MAX precursor. On the other hand, the high intensity (002) peak in the MXene XRD pattern also indicates that the $Ti_3C_2T_x$ flakes tend to align in an orientation parallel to the substrate under the effect of shear force and their own 2D material properties[1]. **b**, Photographs of stable dark-green MXene colloidal solution, exhibiting Tyndall effect. **c**, Optical image of the as-formulated viscous additive-free $Ti_3C_2T_x$ aqueous ink, exhibiting a high viscosity without any solid aggregates and impurities. Inset: the $Ti_3C_2T_x$'s atomic structure. **d**, Dynamic light scattering (DLS) intensity distribution showing the hydrodynamic diameter distribution in the MXene inks. **e**, SEM image of the sediment after etching, showing MAX phase and unexfoliated $Ti_3C_2T_x$ on a SiO_2 wafer. Scale bar, 1 μm. **f**, SEM image of single-layer $Ti_3C_2T_x$ flakes on a porous anodic aluminium oxide (AAO) membrane. Scale bar, 1 μm. **g**, The distribution of the number of layers per laminate <*N*> of the

obtained $Ti_3C_2T_x$ nanosheets. A total of 100 nanosheets from AFM measurements were considered for the statistics of <*N*>. Obviously, the proportion of single-layer nanosheets (~1.5 nm thickness) dominates (>90%) over multi-layer nanosheets in the as-formulated MXene inks. **h**, Size distribution histogram of the obtained $Ti_3C_2T_x$ nanosheets from SEM statistics. The average flake size in $Ti_3C_2T_x$ ink is about 1.6 μm.

In **d**, the red and blue lines represent the MXene inks obtained at two different centrifugation rates, respectively. Since relatively small or large MXene flakes are removed by centrifugation in prior to the ink formulation, the obtained MXene inks (red line) possess a narrower particle size distribution compared to the ink obtained using a previous recipe[2-5]. Combining **g** and **h**, the higher portion of predominantly single-layer, larger flakes in our inks suggest much higher viscosity, storage modulus (G') and loss modulus (G") compared to those of traditional inks, all of which are highly preferred in the extrusion printing for fine lines or structures. Also, these improved rheological properties are expected to enhance the electrical properties of the MXene ink, boost the extrusion printing precision, and prevent oversized nanosheets from accumulating and clogging the nozzle during extrusion printing, which will be discussed in detail later.

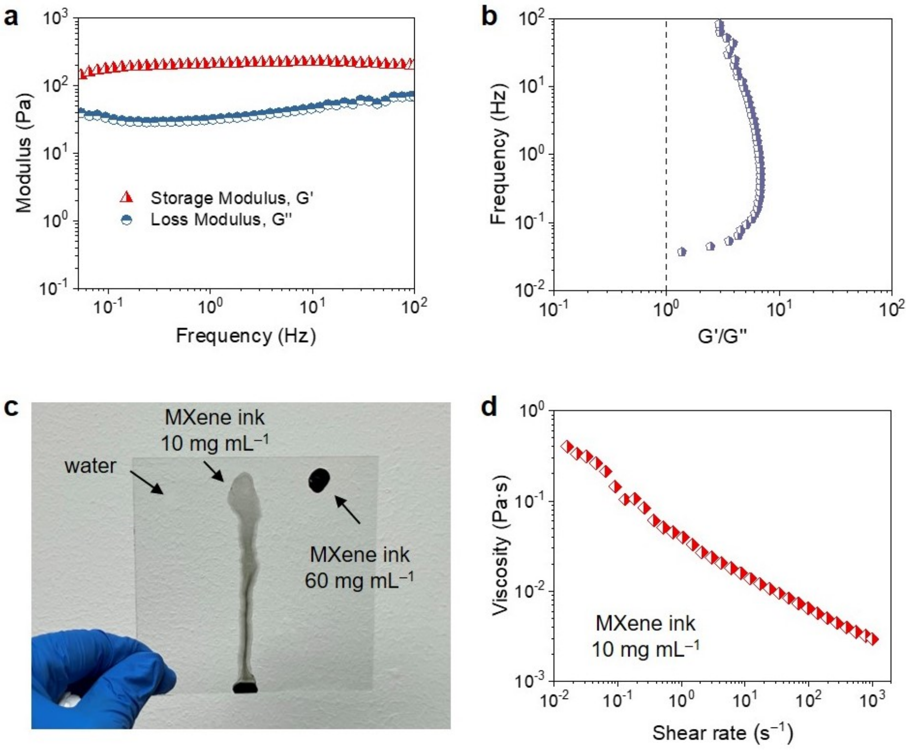

Supplementary Fig. 3 | Rheological properties of MXene aqueous inks. a, The storage modulus (G′) and loss modulus (G″) of MXene ink as a function of frequency. The MXene ink exhibits an obvious dominance of the G′ over G″ in the entire frequency range, suggesting its excellent stability and typical elastic solid properties. **b**, Frequency dependence of the G′/G″ratio in MXene ink. The right side of the dashed line (G′/G″ ratio = 1) suggests the rheological gel-like properties of MXene ink, suggesting its ability to restore the original elastic modulus after exiting the nozzle, thereby maintaining the post-printing shapes after removing the shear force. **c**, Droplet tests of MXene aqueous inks and water on a vertical glass substrate. $Ti_3C_2T_x$ flakes' concentration and sizes determine the ink viscoelastic properties. High-concentration MXene ink has better shape retention ability due to higher storage modulus (G′), allowing it not to flow down on a vertical substrate. **d**, Low-concentration MXene ink (10 mg mL^{-1}) showcases low viscosity, which is not suitable for high-precision extrusion printing.

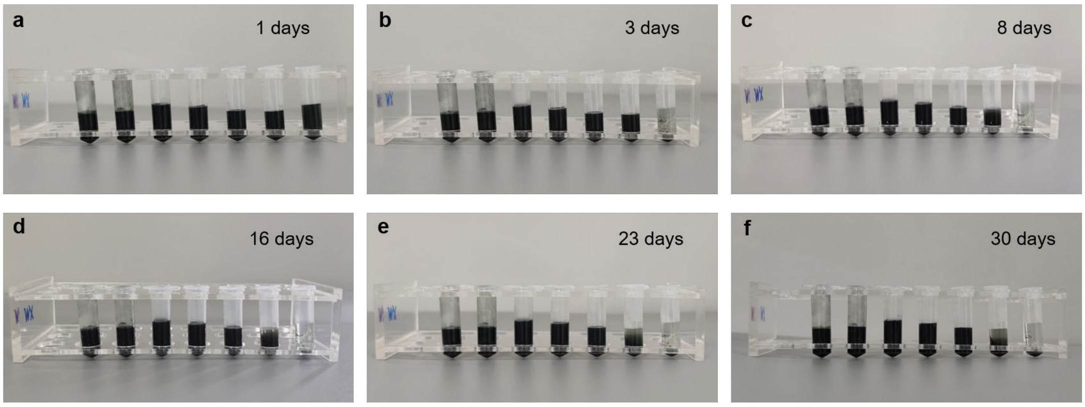

Supplementary Fig. 4 | Long-term testing of MXene aqueous ink stability under ambient conditions. a-f, Seven inks with concentrations (left to right: 60 mg mL^{-1}, 40 mg mL^{-1}, 20 mg mL^{-1}, 10 mg mL^{-1}, 5 mg mL^{-1}, 1 mg mL^{-1}, 0.2 mg mL^{-1}) were configured, and the stability was observed for one month. Here, N_2 was used to eliminate dissolved oxygen in the water to avoid the oxidation of the nanosheets. During a month of observation, the most obvious phenomenon is the aggregation and restacking of MXene flakes due to van der Waals forces in low concentration inks. However, this phenomenon does not occur in high concentration inks because of the high solid content. In addition, no apparent oxidation phenomenon is found in these inks with different concentrations, that is, no the formation of cloudy-white colloidal solutions containing primarily anatase (TiO_2). In conclusion, the developed MXene aqueous inks still have good stability under ambient conditions.

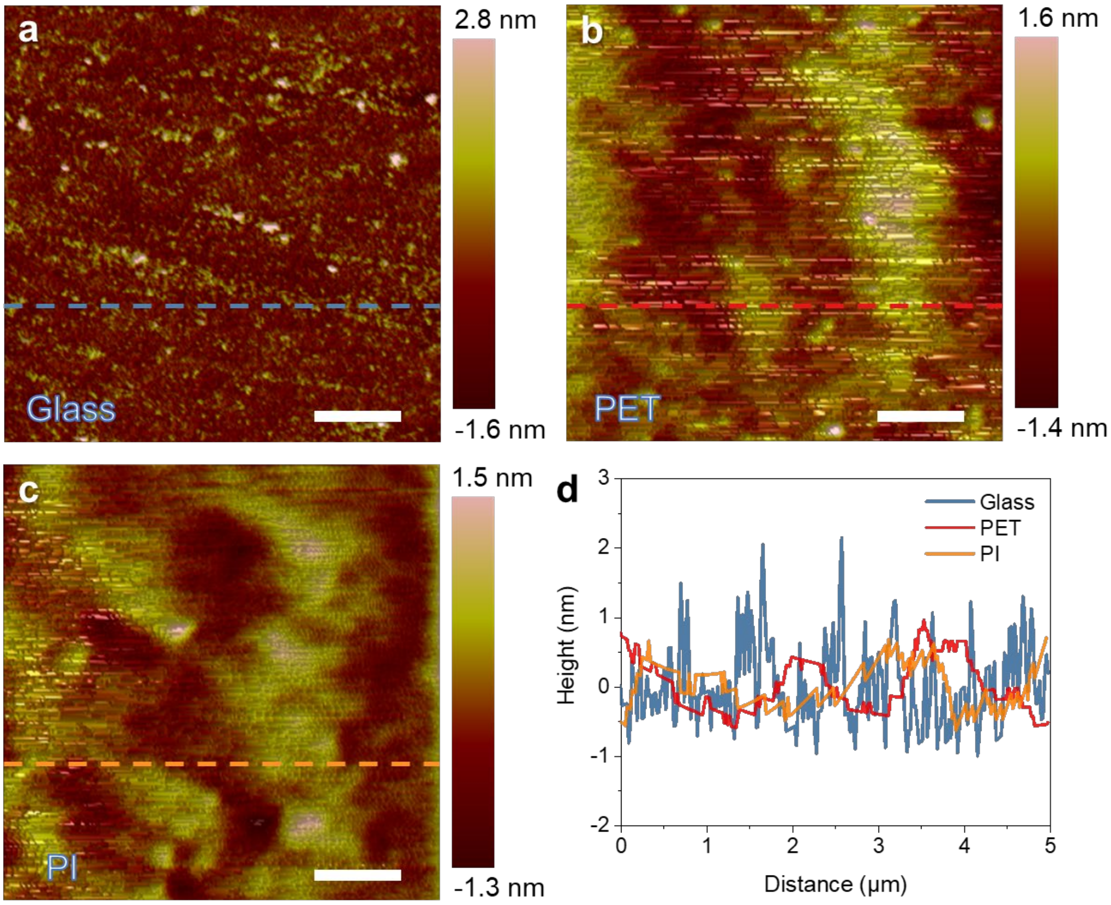

Supplementary Fig. 5 | AFM measurement of substrate surface morphology and roughness. **a-c**, AFM images of glass, PET, and PI substrates used for extrusion printing, respectively. **d**, Height profile along the dashed line of different substrates. These commonly-used printing substrates were demonstrated to have nano-level smooth surfaces. Scale bar, 1 μm.

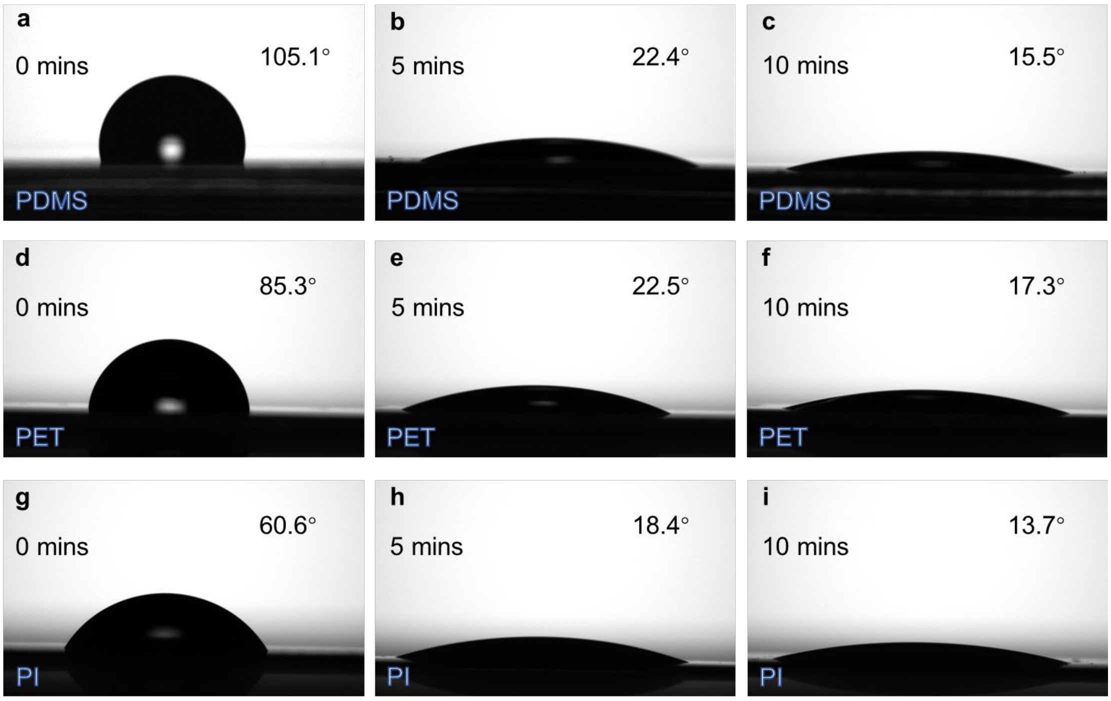

Supplementary Fig. 6 | The wetting property evaluation of three commonly-used polymer substrates after plasma treatment. In general, a small contact angle (≪90°) indicates good wetting, which is beneficial for adhesion between the ink and substrates and forming continuous films. The contact angles between water and **a-c**, PDMS, **d-f**, PET, and **g-i**, PI decrease with increasing plasma treatment time, suggesting the changes in surface energy and the improvement in wetting properties.

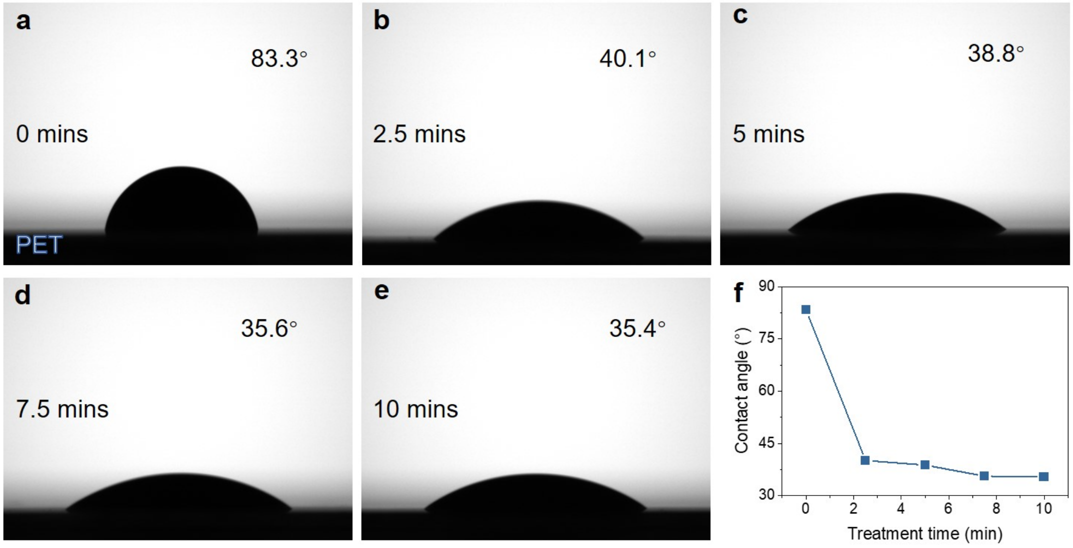

Supplementary Fig. 7 | The wetting property evaluation of MXene aqueous ink on PET after plasma treatment. a-e, The contact angle between MXene ink and PET substrate under different plasma treatment time. **f**, The contact angle between MXene ink and PET substrate as a function of the plasma treatment time.

In general, the commonly-used substrate surface is flat and smooth, yet their hydrophobic surfaces challenge the printing of uniform lines due to the tendency to retract and aggregate in the aqueous MXene inks. Therefore, it is necessary to use plasma technology to improve the wetting behavior of substrate surface. Here, we selected 5 min of plasma time for the substrate pretreatment.

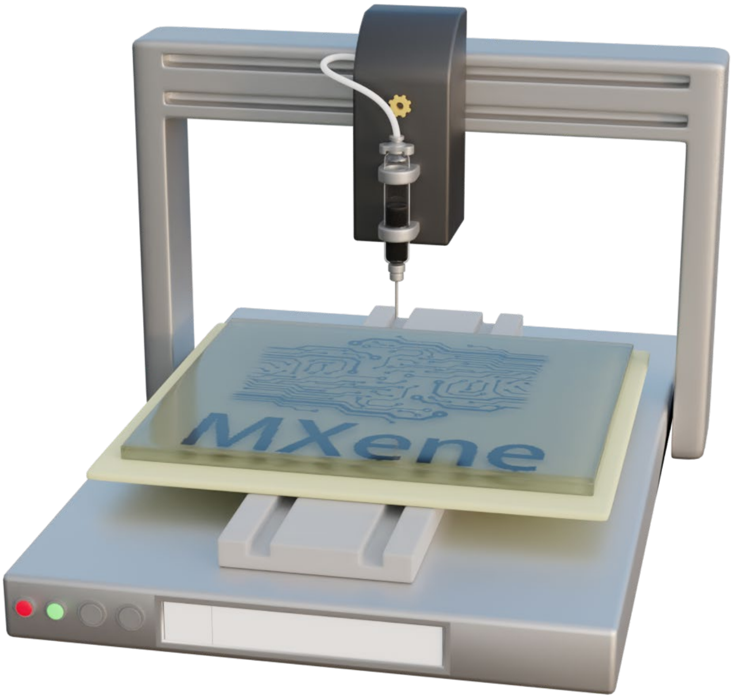

Supplementary Fig. 8 | Schematic illustration of the high-precision robotic deposition system for the direct printing of MXene inks. The three-axis mechanical system is capable of carrying the pneumatic extrusion nozzle according to the preset program to create conductive patterns on specific substrate surfaces accurately. The printing on curved surfaces is performed with the help of a computer-controlled image recognition system.

Unlike conventional subtractive manufacturing (such as photolithography), printing-based additive manufacturing is cost-effective for the rapid, large-scale production of flexible electronics due to its relatively simple procedures and desirable material utilization. Among various printing methods, extrusion-based direct ink printing offers greater opportunities for ink material selection and printing extensibility from micro to macroscale, plane to three-dimensional. Take the comparison with inkjet printing as an example, extrusion printing has apparent advantages in ink viscosity, printing throughput, and film thickness, as mentioned in our previous property-process

guidelines summarized for MXene processing. Extrusion printing can directly deposit the continuous viscoelastic ink filaments without additional masks and accessories, which is a versatile method to realize functional patterns on different substrates (whether flat or curved) under ambient conditions. When considering the increasing structural complexity of flexible electronics, extrusion printing also has advantages in achieving high-precision conformal printing and multi-module integrated manufacturing to avoid time-consuming cumbersome transfer and assembly processes. More detailed comparisons of these two printing techniques can also be found in another of our reported work[3]. On the other hand, our extrusion printable MXene inks have high FoM values to achieve high-efficiency printing. Based on the above advantages, we chose the combination of extrusion printing and additive-free MXene inks to manufacture flexible wireless electronics and also further demonstrated the great potential of this approach in this field.

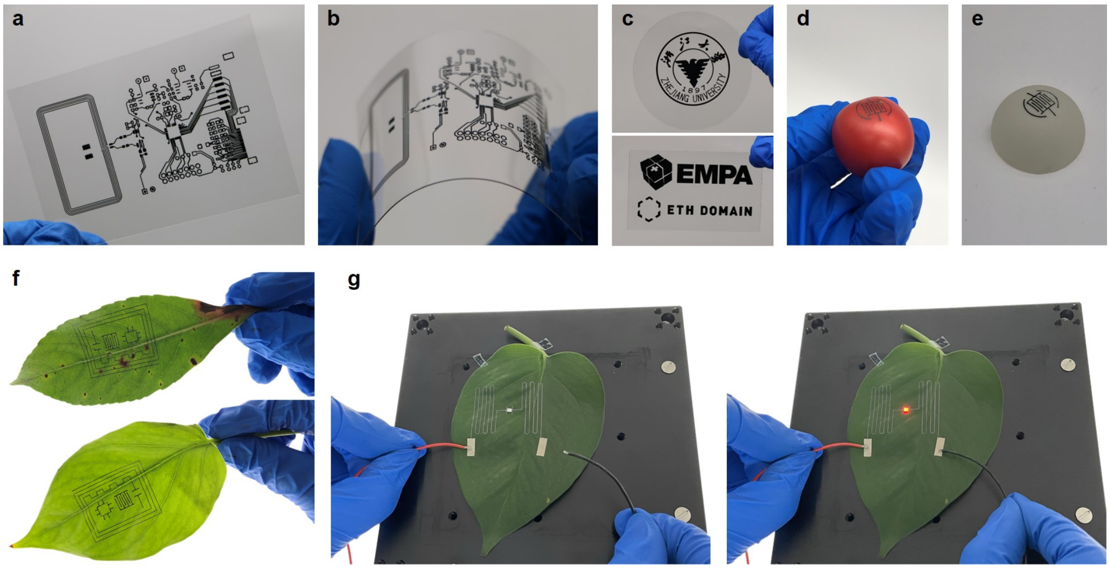

Supplementary Fig. 9 | MXene printed flexible conductive circuits on planar and irregular surfaces. **a-c**, Photographs showing different high-resolution MXene printed circuits and patterns on planar PET substrates, showing good mechanical flexibility. **d-f**, MXene printed circuits on spherical surfaces (tomato and half a ping pong ball, **d** and **e**) and irregular leaf surfaces (**f**). **g**, Conformal printing of conductive MXene circuits on the leaf surface to power a red LED.

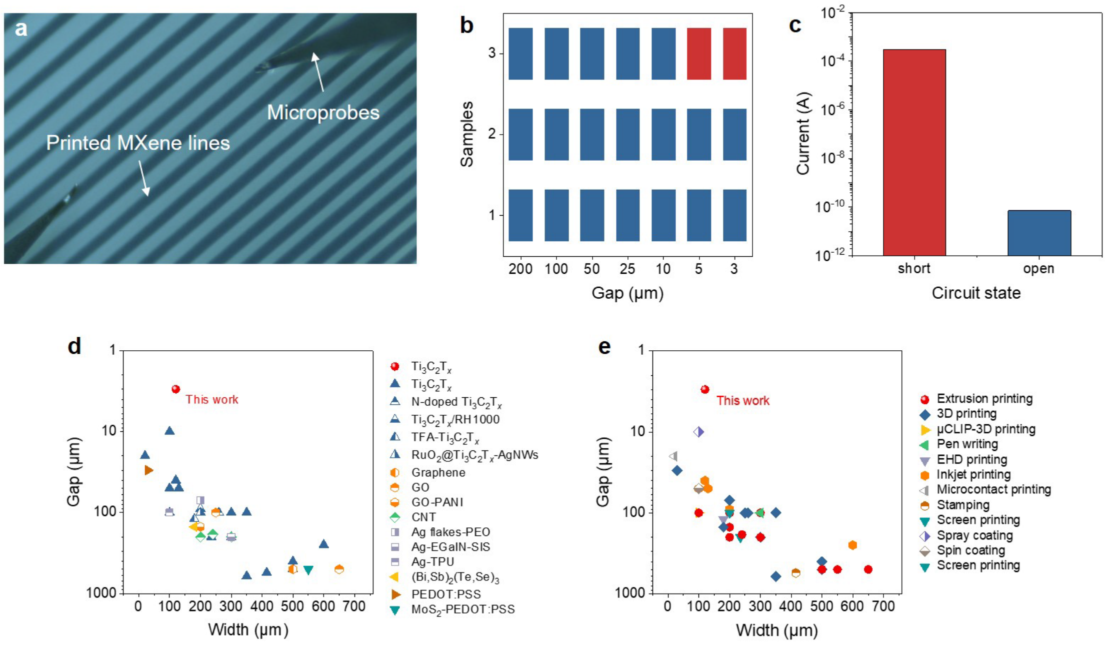

Supplementary Fig. 10 | The electrical measurement of the printed MXene lines with different gaps. a, The continuity test between printed MXene lines was performed on a parameter analyzer with a probe station. **b**, The results of the continuity test. The blue square indicates an open circuit between adjacent lines, and the red one means that the adjacent lines are connected. Three samples in each gap were tested. **c**, The measured current between adjacent printed MXene lines. Printed lines that are too close to each other will cause a larger current due to short circuits. This measurement is performed by applying a voltage of 3 V between the two microprobes. **d**, **e**, The comparison of reported printing resolution of conductive functional ink (different materials **d** and printing methods **e**). In contrast, our work based on MXene inks exhibits obvious advantages in printing resolution, especially the extremely narrow gap width of 3 μm, which has great potential in the manufacture of high-resolution, high packing density electronics. For more details, please refer to Supplementary Table 1.

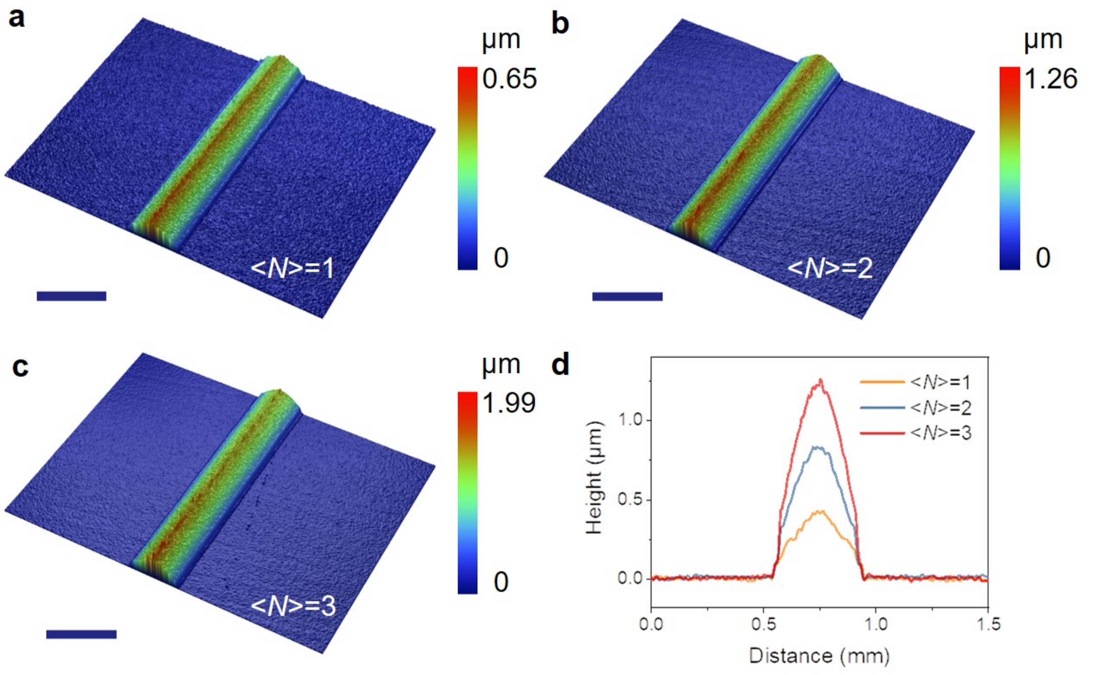

Supplementary Fig. 11 | Height profiles of printed MXene single lines. a-c, Height profiles of printed MXene single lines with various <*N*> from 1 to 3 measured by an optical profilometer. Scale bar, 0.5 mm. **d**, The cross-sectional height profile distribution of printed MXene single lines with different <*N*>. The printed MXene lines have sharp edges, which is the prerequisite for forming high-resolution printed tracks.

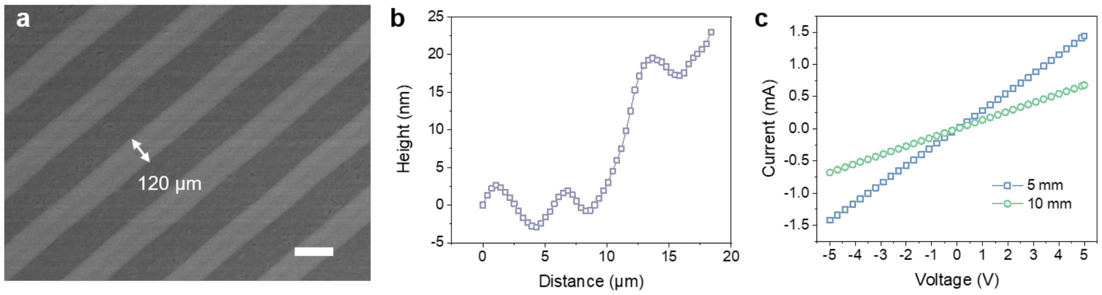

Supplementary Fig. 12 | Further reduction of printed line width. a, SEM image of the printed MXene lines on PET (<*N*>=1). Scale bar, 200 μm. **b**, Height profiles of the printed MXene lines on PET. **c**, Current-voltage plots of the printed MXene lines with different lengths. Considering the potential applications in microelectronics, we try to reduce the printed line width. We demonstrate that the printed MXene line widths can be further reduced to 120 μm with an average height of ~ 20 nm when using custom needles (inner diameter 50 μm) and reconfigured MXene inks (15 mg mL^{-1}). Further, electrical measurements prove these lines are continuous and conductive. The size of the extrusion needle dominates the width of the printed line, and narrower print line widths can be achieved by using smaller needles. However, when using smaller needles, it is also necessary to configure inks with suitable rheological properties, including control of flake size to prevent clogging.

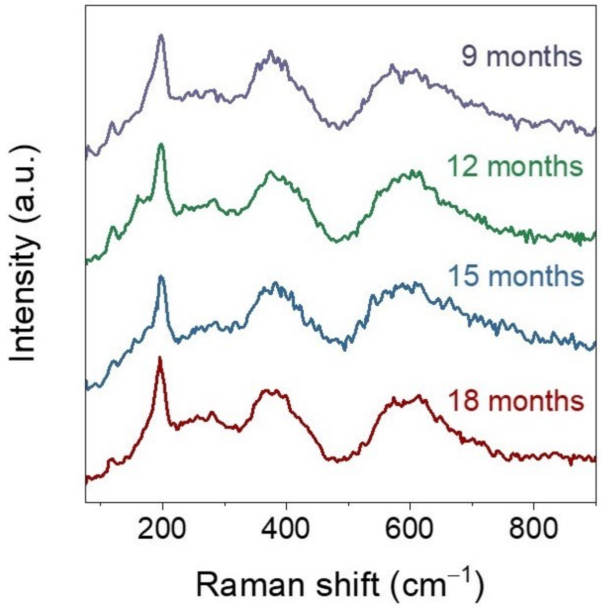

Supplementary Fig. 13 | The stability measurement of printed MXene lines using Raman spectroscopy. The MXene Raman spectra on PET are almost identical after preservation for more than ten months at room temperature, suggesting the as-printed MXenes have well preserved their intrinsic properties without apparent oxidation alongside outstanding suitability on low-cost substrates.

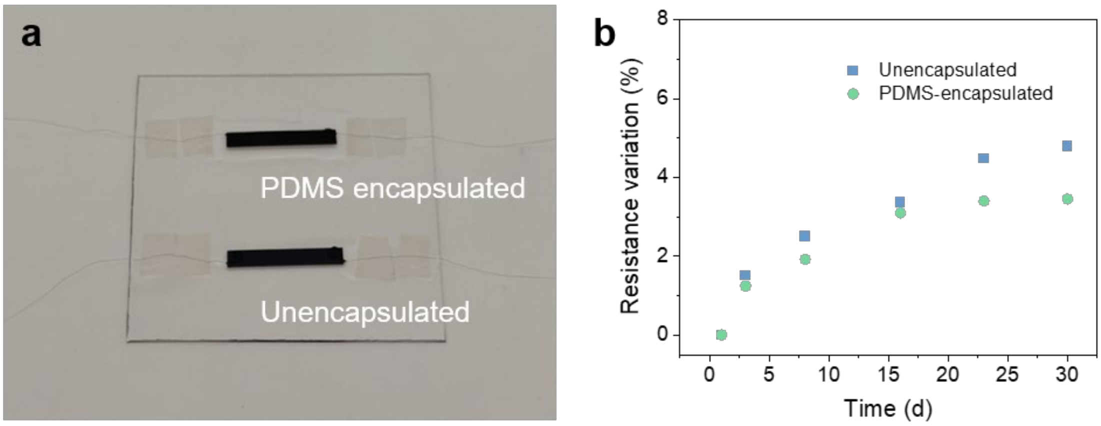

Supplementary Fig. 14 | Long-term stability of printed MXene films under ambient conditions. **a**, Photograph of PDMS-packaged and unpackaged printed MXene films. **b**, The resistance change of the printed MXene films in one month under ambient conditions. After printing, printed MXene films assembled and restacked from MXene flakes can maintain their high conductivity and mitigate oxidation[6]. Both PDMS-encapsulated and unencapsulated printed MXene films maintain their properties well under conditioned storage for one month.

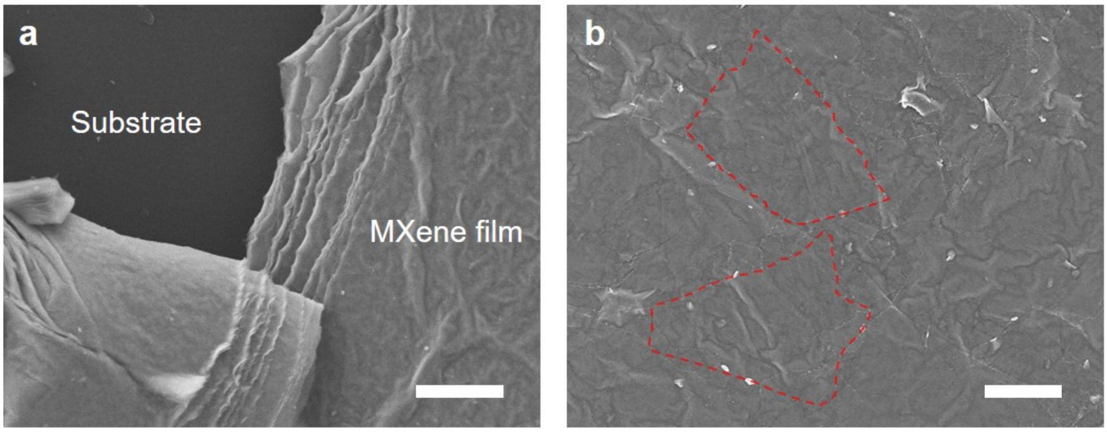

Supplementary Fig. 15 | Microscopic surface morphology of the printed MXene film. a, A cross-sectional SEM view of the printed MXene film on PET. Scale bar, 20 μm. **b**, SEM image shows densely stacked MXene flakes on the surface of the printed MXene film. Scale bar, 500 nm. The cross-section and top view demonstrate the continuous coverage of the printed MXene film on the substrate without protruding flake corners. Due to the shear force during extrusion printing and the 2D material properties of MXene, the $Ti_3C_2T_x$ flakes prefer alignment parallel to the substrate after printing, resulting in dense and highly conductive films. According to recent research, the fast charge transport within the printed MXene films is dominated by the intrinsic intra-flake processes[7].

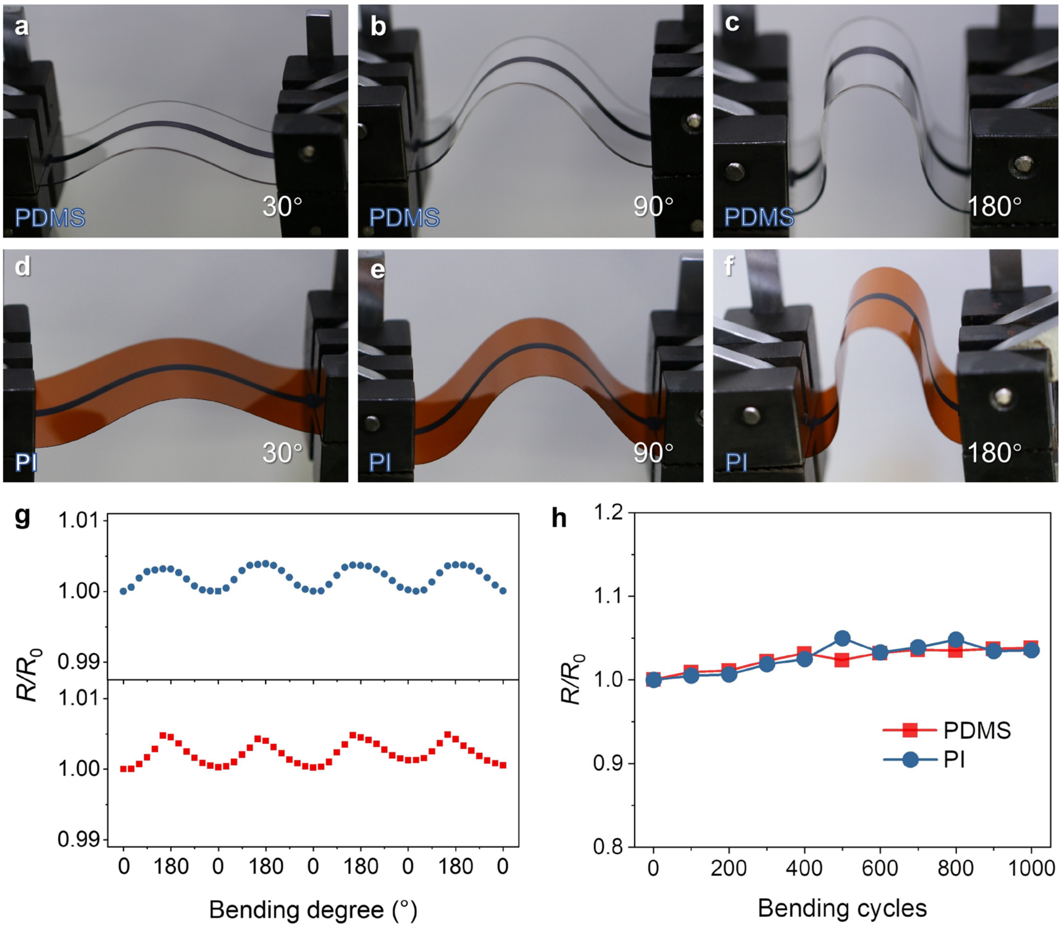

Supplementary Fig. 16 | Bending test for printed MXene lines. Optical photos of printed MXene lines bent at different angles on **a-c**, PDMS and **d-f**, PI, respectively. The printed MXene lines exhibit excellent mechanical flexibility without any cracks during the bending test. **g**, The resistance change of the MXene printed lines (2 mm × 70 mm) at the cyclic bending test from 180° to 0° on PI (blue dots) and PDMS (red dots), respectively. **h**, Long-term bending test for printed MXene lines on PDMS and PI. The resistance of the MXene line measured on PDMS or PI after different bending times was normalized to the initial resistance before bending. Although repeated bending slightly increases the resistance of printed MXene lines upon bending and decreases the numbers upon releasing, the tight adhesion of the MXene nanosheets to substrate provides great mechanical stability, thereby maintaining the resistance change rate within 5% over 1,000 bending cycles.

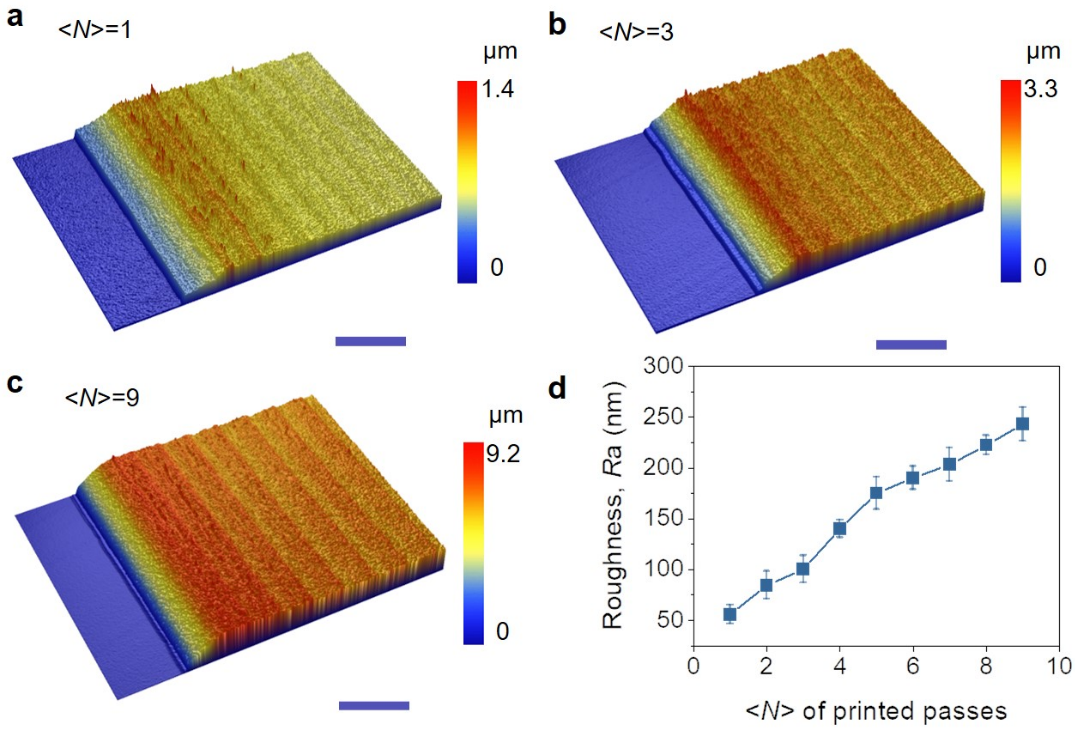

Supplementary Fig. 17 | The morphologies of the printed MXene films. a-c, Height profiles of printed MXene films on PET substrate with different <*N*> measured by an optical profilometer. Scale bar, 0.5 mm. As the <*N*> increases from 1 to 9, the thickness linearly boosts from 0.82 to 6.73 µm, and the as-printed edges are sharp. **d**, The surface roughness of printed MXene films plotted as a function of <*N*>. In general, the surface roughness increases as <*N*> increases, but even when <*N*>=9, the value of *R*a is still far less than 1 µm, suggesting the smooth surface in the as-printed lines.

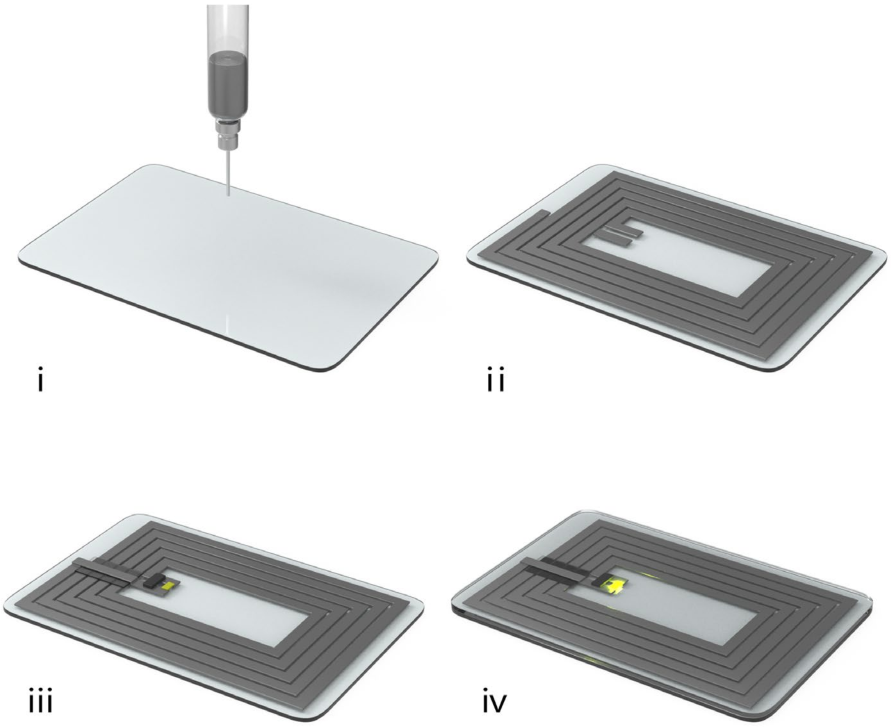

Supplementary Fig. 18 | Schematic illustration of the fabrication process of MXene printed NFC tags. Firstly, the printing robot arm moves the printing head to complete the printing of the antenna coils on flat substrates (300 μm PDMS, supported by PET) according to the pre-trajectory and program. Later, a polyurethane (PU) insulating film is applied onto the overlapping area between the top interconnect and the bottom antenna coil, then a conductive MXene path is printed on it. An NFC chip (NTAG 215 or FM11RF08) and a LED were mounted onto the antenna terminals with conductive MXene clays to yield conductive joints without using other conductive adhesives. Finally, a 200 μm PDMS encapsulation layer was used to complete the fabrication of flexible NFC tags.

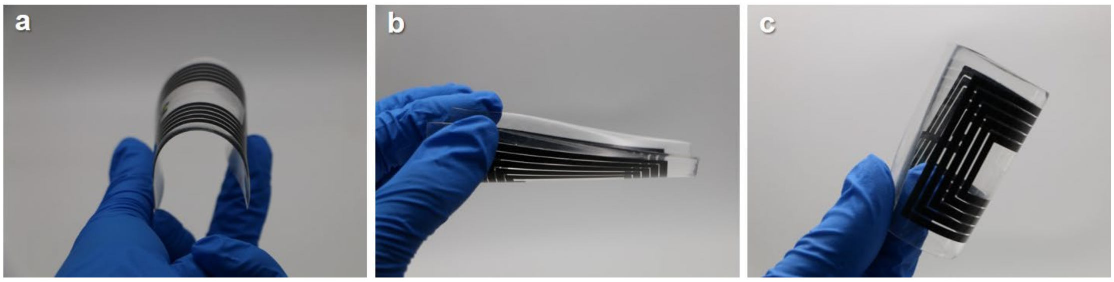

Supplementary Fig. 19 | Flexible MXene printed NFC tag. Photographs showing the excellent flexibility of MXene printed NFC tag on PDMS substrate. The stored ZJU website messages in MXene NFC tag can be accessed via a nearby NFC-enabled smartphone. Even under bent or twisted at large angles, the MXene NFC tag can still work normally.

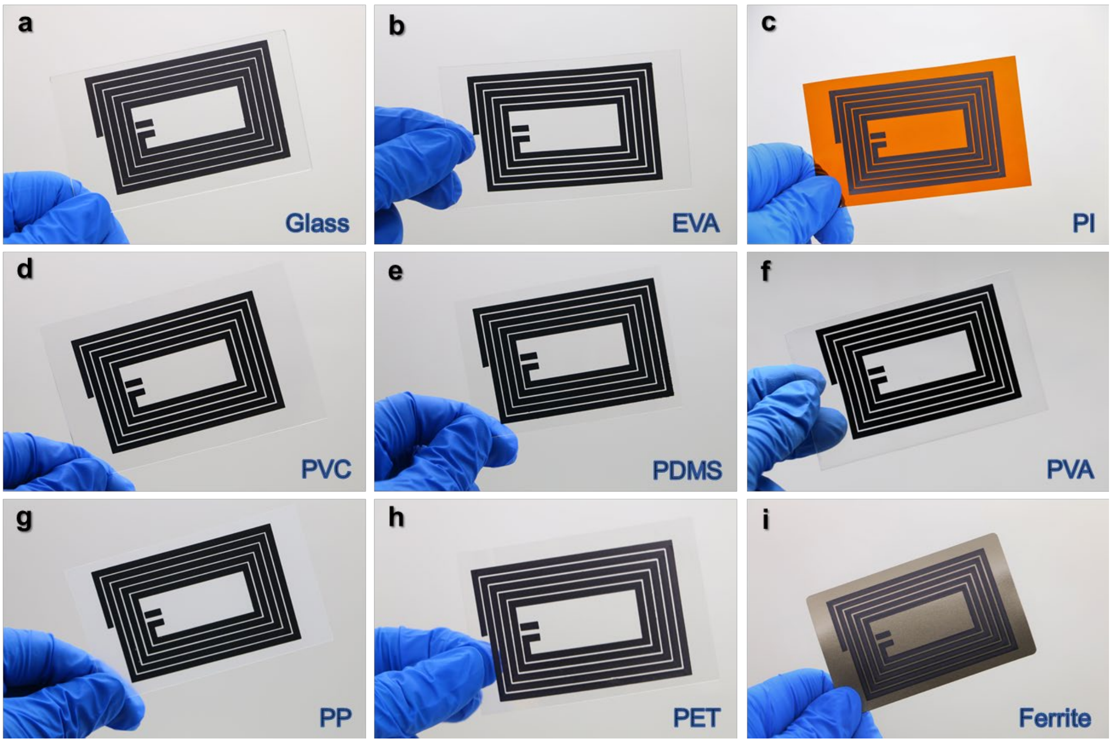

Supplementary Fig. 20 | Diversity of printing substrate selection. Photographs showing the production of printed MXene antennas on different substrates. The variety of printing substrates for choice can meet various functional needs, such as the choice of ferrite substrate enabling the fabricated NFC tags to resist the magnetic interference of metal.

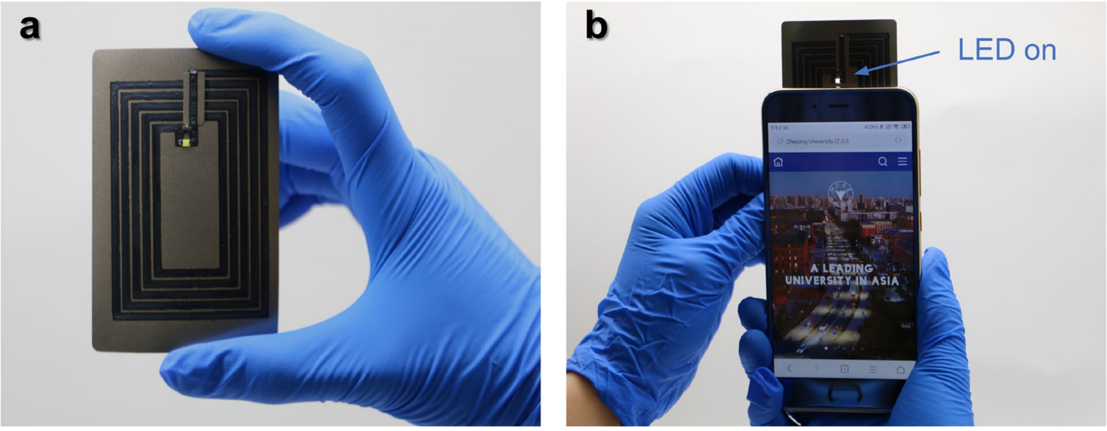

Supplementary Fig. 21 | Unencapsulated MXene printed NFC tag on ferrite substrate. The unencapsulated NFC tag (<*N*> = 15) was found to be normally operable after being stored in low humidity condition for nearly two years. **a**, Optical image showing the unencapsulated NFC tag. **b**, The LED indicator on the NFC tag will still light up when the smartphone is near. The ZJU website information stored in the NFC tag can be recognized by the smartphone.

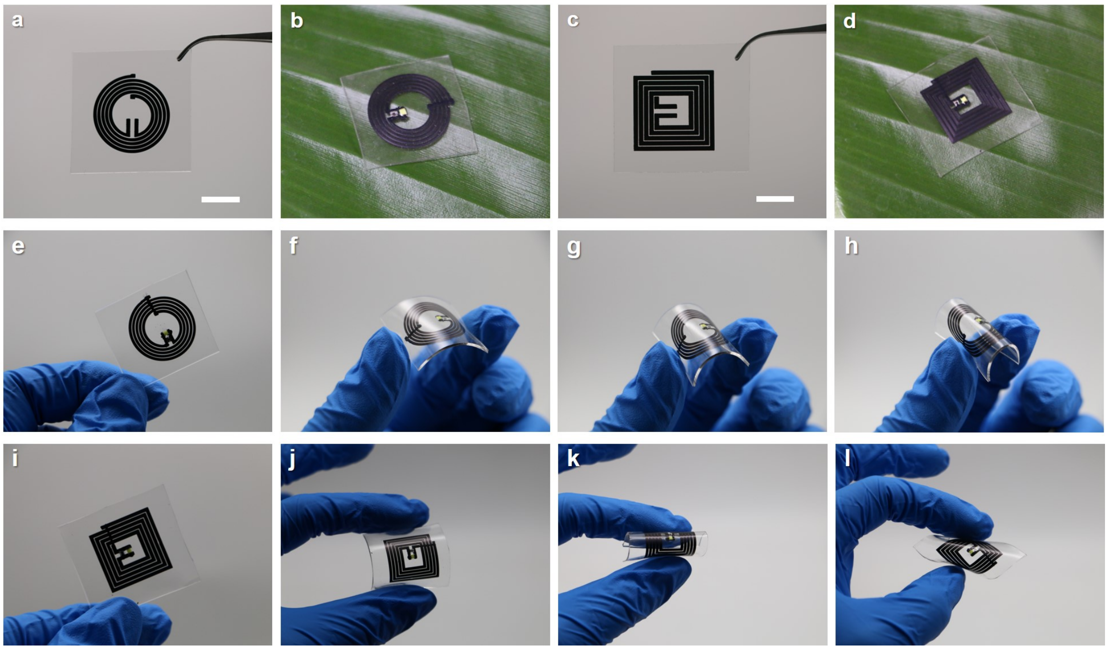

Supplementary Fig. 22 | Small-sized MXene printed NFC antenna tags. Photographs showing small-sized **a, b** circular and **c, d** square MXene printed NFC antenna coils (left) and functionalized tags (right) on PDMS. Scale bar, 10 mm. These small-sized tags show excellent flexibility, as shown in **e-h** (circular tags) and **i-l** (square tags).

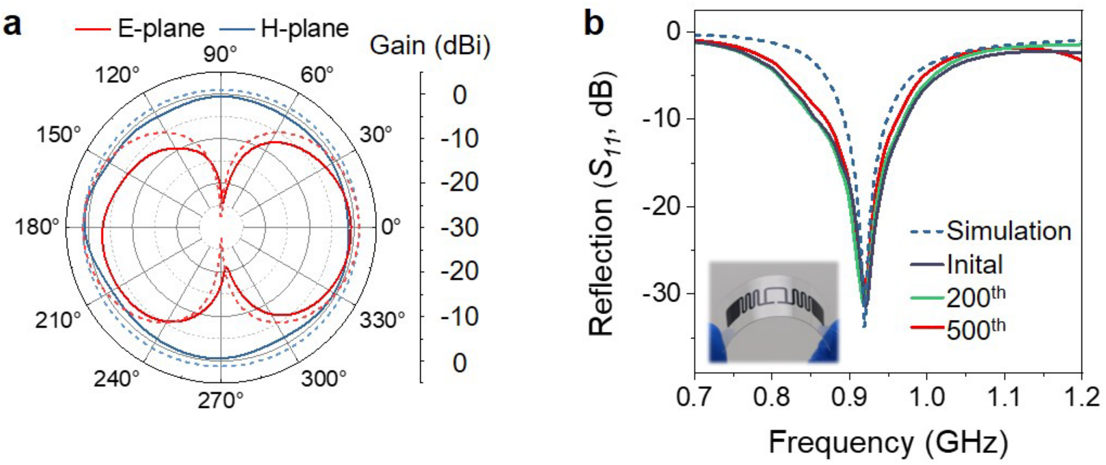

Supplementary Fig. 23 | Electromagnetic characteristics of the MXene dipole antenna. a, The measured 2D polar radiation pattern of the MXene dipole antenna at 920 MHz. The solid and dashed lines represent the results from measurement and simulation, respectively. **b**, Return loss |S11| spectra for the flexible MXene dipole antenna with different bending numbers. The inset shows the bending state of the MXene dipole antenna on PET.

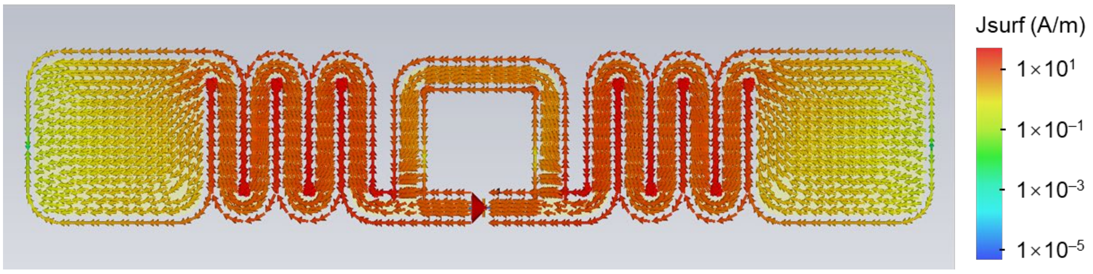

Supplementary Fig. 24 | Current distribution of the MXene dipole antenna at 920 MHz. Identifying the current distribution is important for further antenna optimization, as the high current density area is sensitive to impedance tuning. This simulation of current distribution was performed by CST software.

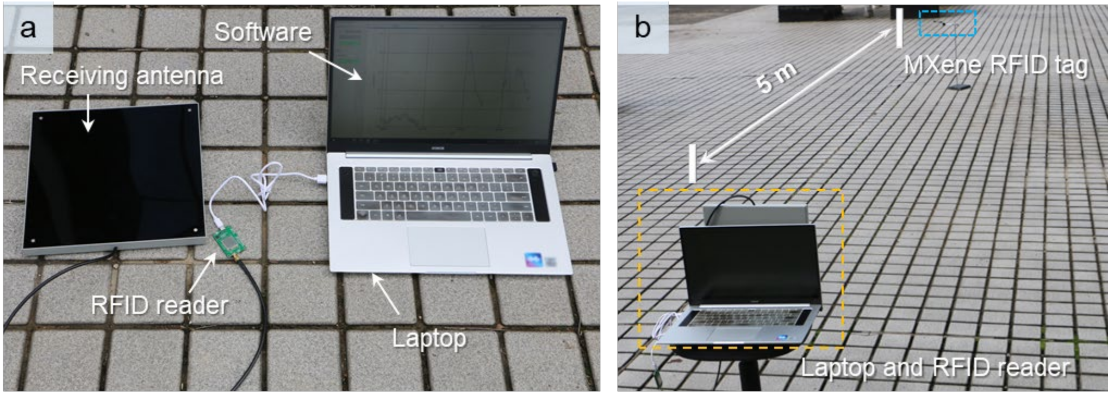

Supplementary Fig. 25 | RFID temperature sensing system. a, Optical image showing the laptop-connected RFID reading system and the custom software for temperature monitoring. **b**, Optical image showing the operation mode and the maximum reading range of the MXene RFID temperature tag. The RFID reader is responsible for sending signals and transmitting energy to the dipole antenna, as well as receiving the returned data. The RFID chip (NMV2D CAB0) has a built-in sensor, endowing the ability of temperature monitoring. Custom computer software can display the temperature data in real-time, allowing multiple tags to work simultaneously. The entire RFID reading system is simple in architecture and easy to operate. The MXene RIFD tag has a far reading range and a long working life, as it has been performing well in the laboratory for more than a year.

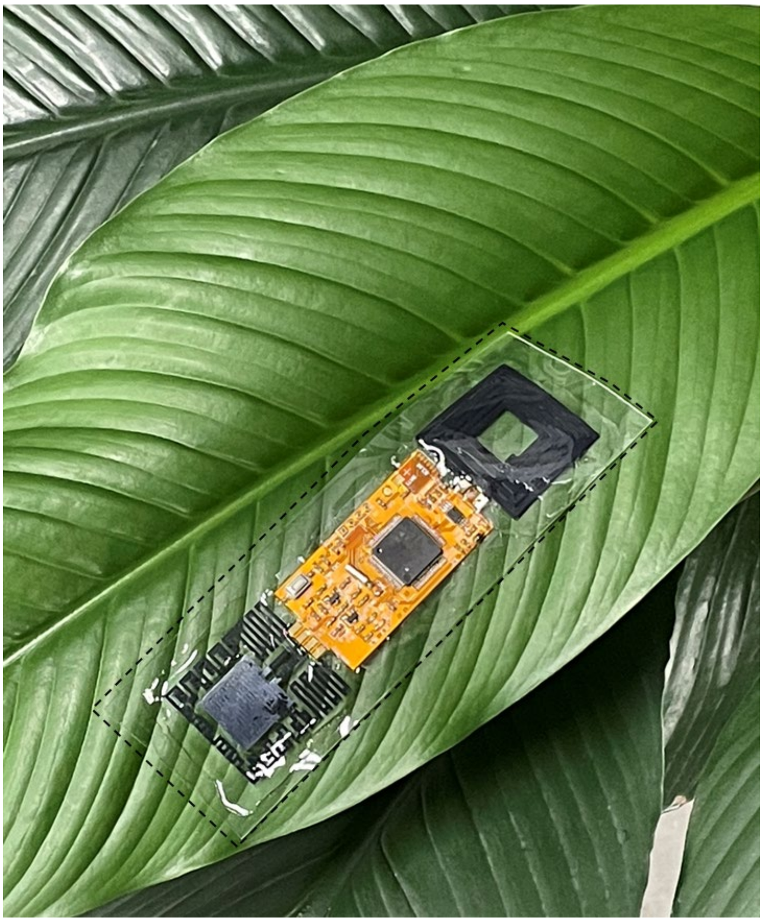

Supplementary Fig. 26 | The photo of all-MXene-printed flexible NFC-enabled integrated sensing system. This system consists of an external flexible printed circuit board (FPCB) and three all-MXene-printed modules (wireless, energy, and sensing) on PDMS.

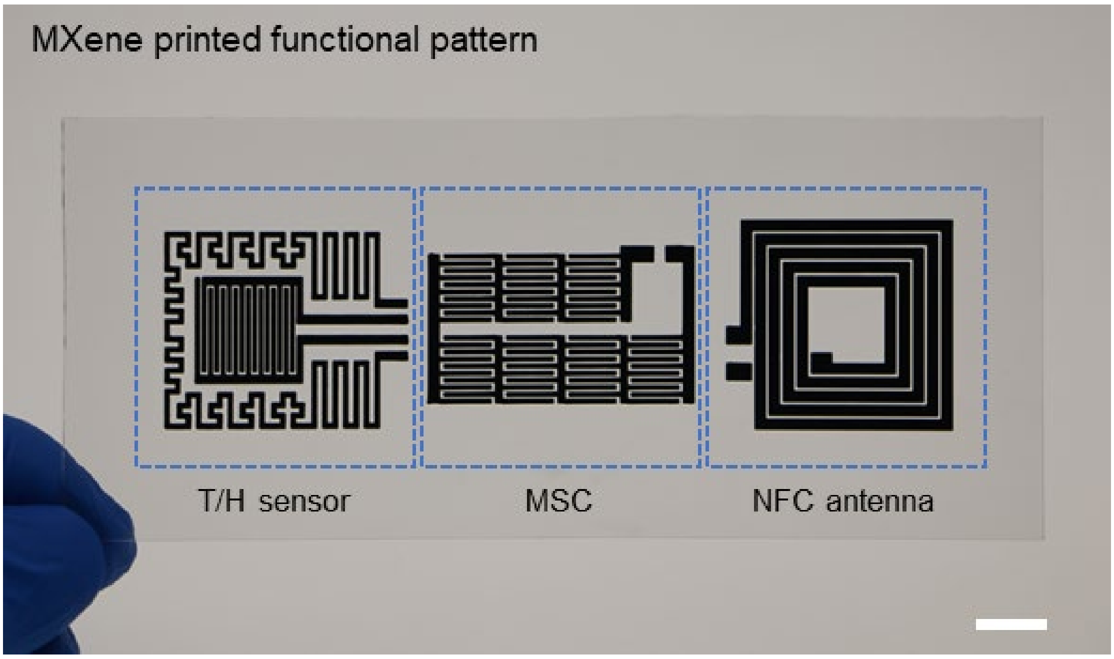

Supplementary Fig. 27 | All-MXene-printed functional pattern for NFC-enabled integrated electronics. Photograph shows a top view of the all-MXene-printed functional pattern on PDMS, which consists of three functional modules, including a T/H sensor for ambient sensing, a MSC module for energy storage, and an NFC antenna for transmitting information and obtaining power. Scale bar, 10 mm.

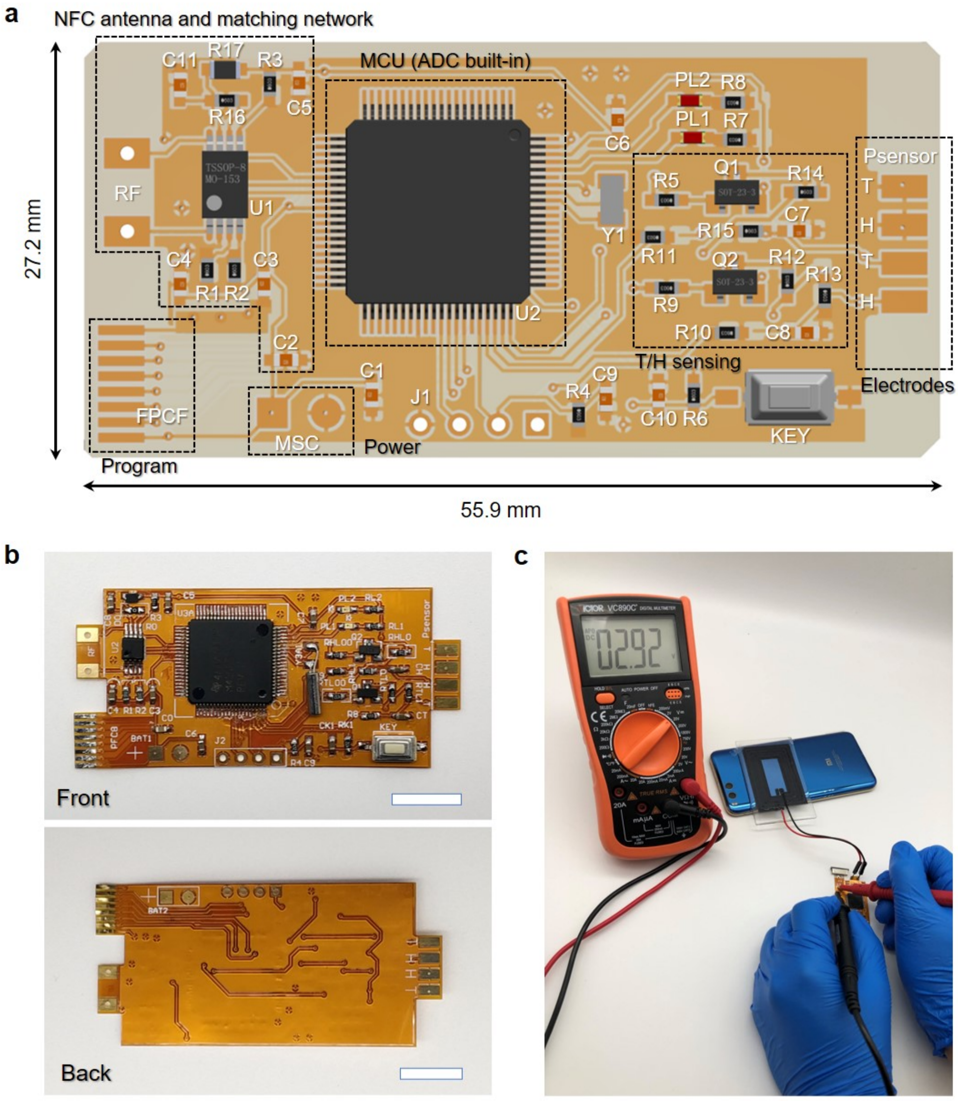

Supplementary Fig. 28 | Custom flexible printed circuit board (FPCB) for the all-MXene-printed NFC-enabled integrated electronics. a, Schematic diagram shows the top view of the custom circuit board with dimensions marked. The functional blocks are highlighted in the dotted box. The corresponding components are detailedly shown in Supplementary Table S5. **b**, Optical images of the front and back of the custom flexible printed circuit board fabricated on PI. Scale bar, 10 mm. The FPCB is used as a control module to connect three MXene printed modules. **c**, The NFC chip can provide a stable voltage output (~3V) to power the MCU for T/H sensing and charge the energy module. This system supports setting the data sampling time interval in the case of long-term power supply.

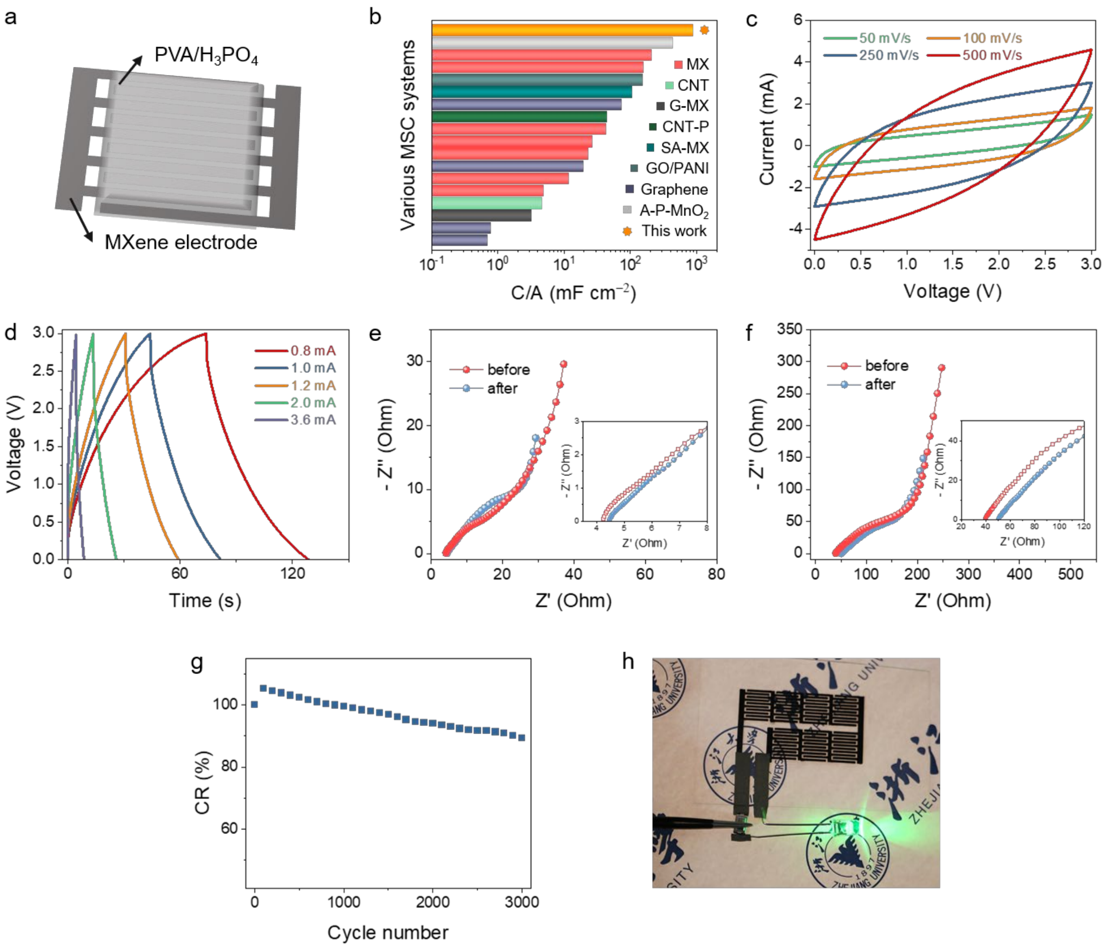

Supplementary Fig. 29 | Electrochemical performance of the planar MXene MSCs. a, The architecture of a MSC unit fabricated by the extrusion printing of the MXene ink. Note that MXene lines function as both current collectors and electrodes; no additional metal current collectors are required. The device was measured in a voltage window of 0.6 V with PVA/H_3PO_4 gel as the electrolyte. **b**, C/A comparison of this work to other printed MSCs (see Supplementary Table 3). **c**, CV curves of the MSC module with scan rates from 50 to 500 mV s^{-1}. **d**, GCD curves of the planar MXene MSC module at an applied current from 0.8 to 3.6 mA. **e, f**, Electrochemical impedance spectroscopy of the MXene MSC (e) unit and (f) module before and after the CV measurements at various scan rates. **g**, Cyclic stability test of the MSC module at an applied current of 7.2 mA. After 3000 test cycles, ~90% of the initial capacitance can be retained, which is enough for the use-case of the microgrid. **h**, Planar MXene MSC module to power a green LED light. When this module

is fully charged, it can support the entire sensing system to continue working for ~12 s. These results demonstrate its ability to meet specific energy needs as a tandem device for flexible electronics.

The CV and GCD measurement were performed in a voltage window of 3 V, which can power most electronic devices. This MXene MSC module consisting of seven MXene MSC units connected in serial allows charge/discharge at a high rate, suggesting the efficient connectivity between MSC units and small equivalent series resistance (ESR) that helps reduce unnecessary energy and power losses during the charge-discharge cycle. Note that by direct printing of MXene inks, a versatile combination (in serial and/or parallel) can be randomly selected to meet the energy/power demands, demonstrating the great feasibility in satisfying various occasions.

Based on the intercept in the high-frequency region, the Nyquist plot shows that the electrolyte resistance of the MSC unit and module are 4.2 Ω and 39.7 Ω, respectively. After repeated CV measurements, both the electrolyte resistances exhibit a slight increase. No high-frequency semicircle is observed, indicating the charge transfer resistance (Rct) was negligible. The EIS measurement demonstrates the pseudocapacitive charge storage behavior, suggestive of capacitive behavior and rapid ion diffusion kinetics in the MXene interdigital electrodes.

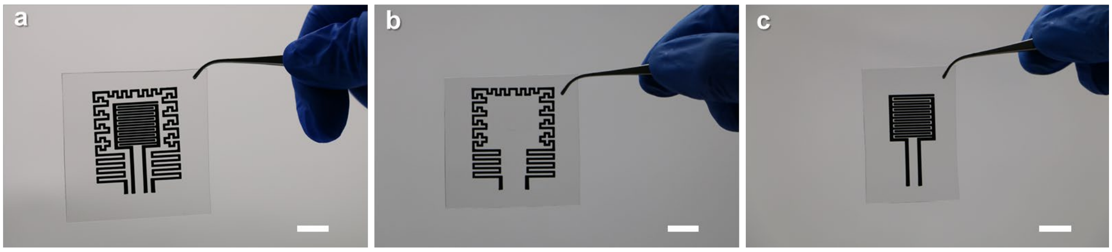

Supplementary Fig. 30 | All-MXene-printed temperature and humidity sensing module. a-c, Digital photographs show the all-MXene-printed functional conductive patterns used for (**a**) T/H sensing module or used only for (**b**) temperature sensor and (**c**) humidity sensor. Scale bar, 10 mm. The MXene channel in the temperature sensor was designed into a symmetrical curved shape to augment resistance in a limited space and enhance sensitivity to temperature changes.

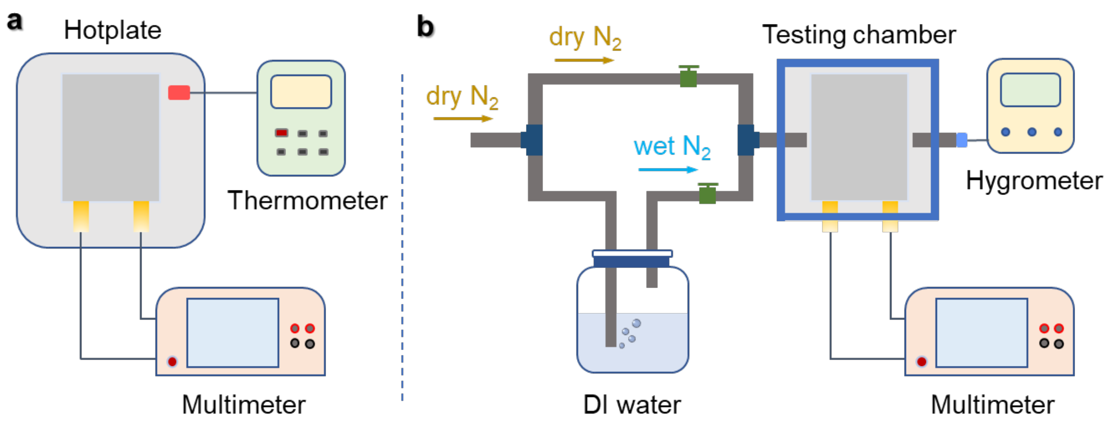

Supplementary Fig. 31 | Characterization of MXene temperature and humidity sensors. Schematic diagrams illustrate the measurement systems for **a**, temperature and **b**, humidity sensing, respectively.

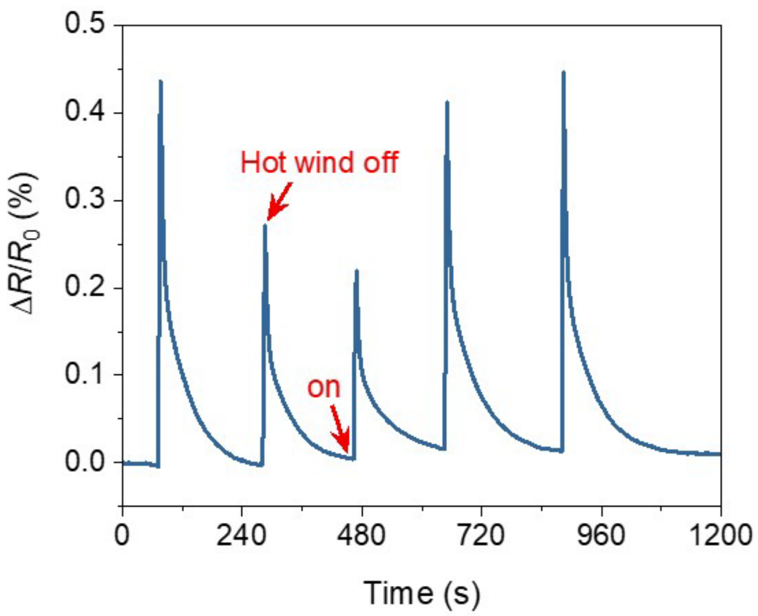

Supplementary Fig. 32 | Temperature sensor for monitoring temperature changes. The prepared MXene temperature sensor exhibits high sensitivity to the temperature change, allowing a keen sense of the start and shutoff of the hot wind caused by an electric hairdryer.

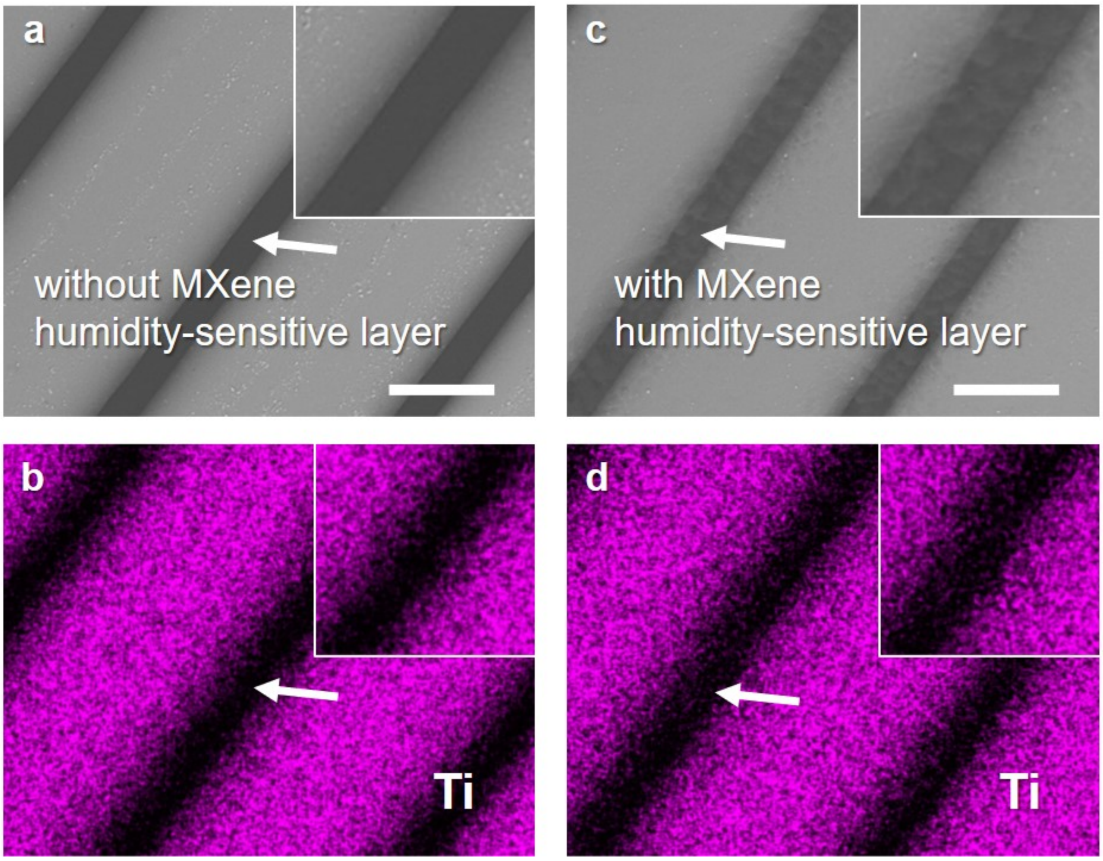

Supplementary Fig. 33 | Characterization of MXene humidity-sensitive layer. SEM images and corresponding EDS mapping images show the morphology of MXene printed interdigital electrodes **a, b** without or **c, d** with MXene film. Scale bar, 400 μm. A layer of MXene thin film covering the interdigital electrode serves as the humidity-sensitive layer. Generally, the humidity sensitivity of the sensors will increase as the thickness of the humidity sensing layer increases, but this also results in an increased response time of sensors[8].

Supplementary Table 1 | Resolution comparison of reported functional ink printing (Fig. S10d,e).

Material	Method	Gap (μm)	Width (μm)	Post-processing	Application	Ref.
$Ti_3C_2T_x$	Extrusion printing	3	120	–	Antenna, MSC, and sensors	This work
$Ti_3C_2T_x$	Extrusion printing	100	200	Substrate heating (60 °C)	MSC	3
N-doped $Ti_3C_2T_x$	Extrusion printing	100	300	Freeze-dried and HI reduction (90 °C, 2 h)	MSC	9
$Ti_3C_2T_x$	3D printing	200	300	Freeze-dried	Battery	10
$Ti_3C_2T_x$	3D printing	500	500	None	MSC	11
N-doped $Ti_3C_2T_x$	3D printing	100	260	Freeze-dried and HI reduction (90 °C, 2 h)	Hybrid capacitor	12
$Ti_3C_2T_x$	3D printing	100	350	Freeze-dried (24 h)	MSC	13
$Ti_3C_2T_x$ -$ZnSO_4$	3D printing	400	500	Freeze-dried	Hybrid capacitor	14
$Ti_3C_2T_x$	3D printing	610	350	Freeze-dried	MSC	15
$Ti_3C_2T_x$	μCLIP-3D printing	100	100	Vacuum preservation	Strain sensor	16
$Ti_3C_2T_x$	Pen writing	100	300	–	MSC	17
TFA-$Ti_3C_2T_x$ (ethanol)	EHD printing	120	180	Vacuum annealed (160 °C, 1h)	TFT	18
$Ti_3C_2T_x$ (NMP)	Inkjet printing	50	130	Substrates heating (60 °C)	MSC	3
$Ti_3C_2T_x$	Inkjet Printing	50	100	Substrates heating (50 °C)	Photonics device	19
$Ti_3C_2T_x$ (DMSO)	Inkjet Printing	40	120	Substrates heating (70 °C)	Electromagnetic shielding	20
$Ti_3C_2T_x$ /PH1000	Inkjet Printing	90	200	Substrates heating (40 °C)	MSC	21
$Ti_3C_2T_x$	Thermal Inkjet Printing	250	600	–	MSC	6
$Ti_3C_2T_x$	Microcontact printing	20	20	Vacuum dried and removal of PDMS stamp	FET	22
$Ti_3C_2T_x$	Stamping	550	415	–	MSC	5
$Ti_3C_2T_x$	Screen printing	200	235	Dried at 80°C	MSC	2
$Ti_3C_2T_x$	Spray coating	10	100	Hot air-dried (50 °C)	MSC	23
$Ti_3C_2T_x$	Spin coating	50	100	Baked (130 °C, 3 h)	Neural probe	24

RuO_2@ $Ti_3C_2T_x$-AgNWs	Screen printing	100	200	Dried under ambient condition	MSC	25
Graphene	Extrusion printing	500	500	Photonic annealing	MSC	26
GO	Extrusion printing	500	650	Substrates heating (70 °C) and HI reduction	MSC	27
GO	3D printing	100	250	Freeze-dried, chemical reduction, and annealing	MSC	28
GO-PANI	Extrusion printing	150	200	Dried overnight (60 °C)	MSC	29
CNT	Extrusion printing	200	300	Dried and conditioned (21 °C, 50% RH)	Strain sensor	30
CNT	Extrusion printing	185	240	Vacuum annealing (130 °C, 12 h)	MSC	31
CNT	Extrusion printing	200	200	Dried in ambient conditions (20 min)	Capacitive sensor	32
Ag flakes-PEO	3D printing	70	200	Ethanol evaporation	Inductive coil	33
Ag-EGaIN-SIS	Extrusion printing	200	300	Solvent evaporation (60 °C, 30 min)	Stretchable circuit	34
Ag-TPU	Extrusion printing	100	100	Cured at 80 °C for 2 h	Strain sensor	35
$(Bi,Sb)_2(Te,Se)_3$	3D printing	150	180	Dried (110 °C, 5 min) and annealed (450 °C, 30 min)	Thermoelectric generator	36
PEDOT:PSS	3D printing	30	30	Dried (60 °C, 24 h) and annealing (130 °C, 1.5 h)	Neural probe	37
MoS_2-PEDOT:PSS	Extrusion printing	500	550	Vacuum dried at 60 °C overnight	Supercapacitor	38

Extrusion printing here refers to planar 2D printing. EHD: electrohydrodynamic; μCLIP: microcontinuous liquid interface production; TFA: trifluoroacetic acid; TFT: thin-film transistor; NMP: N-Methyl-2-pyrrolidone; DMSO: dimethyl sulfoxide; AgNWs: silver nanowires; GO: graphene oxide; PANI: polyaniline; CNT: carbon nanotube; PEO: poly(ethylene oxide); EGaIn: eutectic gallium–indium; SIS: styrene-isoprene block copolymers; TPU: thermoplastic polyurethane; PEDOT:PSS: poly(3,4-ethylenedioxythiophene):polystyrene sulfonate

Supplementary Table 2 | Electrical conductivity comparison of various printable functional inks.

Materials	Solvent	Method	σ, S cm^{-1}	Substrate	Post-processing	Ref.
MXene	water	Extrusion printing	6.9×10^3	PET	–	This work
60 wt % silver nanoflakes	PUA	Screen printing	6.02×10^4	Fabric	UV/20 s for curing	39
56 wt % silver nanoflakes	water	Stencil printing	7.38×10^3	PDMS	80 °C/30 mins for curing	40
80 wt % silver nanoflakes	PDMS	Stencil printing	1.51×10^4	Silicone	160 °C/1 h for curing	41
Zn microparticles	PVP	Screen printing	9.7×10^3	Na-CMC	Laser sintering process	42
Zn microparticles	PVP	Screen printing	3.0×10^3	PLGA	Electrochemical sintering in acetic acid	43
LMPs	n-Decyl alcohol	Screen printing	8×10^3	PDMS	–	44
H-doped LM MPs	toluene	Nozzle printing	2.5×10^4	PDMS	120 °C/3 h for solvent removal	45
AgInGa-SIS	toluene	Extrusion printing	7.02×10^3	SIS	60 °C/1 h	46
LMPs-Cu-SIS	toluene	Screen printing	1.1×10^4	SIS	HCl fume chemical sintering	47
bGaIn	ethanol	Spray printing	2.06×10^4	3M VHB tape	Sintering at 900 °C/ 30 min	48

PUA: polyurethane acrylate; Na-CMC: sodium carboxymethyl cellulose; PLGA: poly(lactic-co-glycolic acid; LMPs: EGaIn liquid metal particles; H-doped LM MPs: hydrogen doping liquid metal microparticles; SIS: styrene-isoprene block copolymers; bGaIn: biphasic Ga-In

Supplementary Table 3 | Simulation results for printed MXene NFC coils with different geometrical parameters (fixed parameter: size = 45 × 70 mm，thickness = 20 μm).

Number of turns	Width (mm)	Spacing (mm)	R (Ω)	L(μHr)	*Q* factor
3	1.5	0.5	55.924	1.644	2.504
	1.5	1	51.632	1.428	2.356
	1.5	1.5	47.581	1.245	2.229
	2	0.5	43.358	1.438	2.825
	2	1	35.948	1.219	2.888
	2	1.5	33.957	1.077	2.701
	2	0.5	30.184	1.203	3.395
	2.5	1	28.626	1.049	3.122
	2.5	1.5	26.170	0.922	3.003
4	1.5	0.5	42.492	1.055	2.115
	1.5	1	38.988	0.956	2.088
	1.5	1.5	37.166	0.866	1.985
	2	0.5	30.458	0.936	2.619
	2	1	28.748	0.851	2.521
	2	1.5	27.117	0.766	2.406
	2.5	0.5	24.148	0.831	2.931
	2.5	1	22.646	0.756	2.843
	2.5	1.5	21.208	0.694	2.789
5	1.5	0.5	82.799	2.436	2.507
	1.5	1	60.402	1.867	2.633
	1.5	1.5	56.200	1.583	2.400
	2	0.5	49.520	1.867	3.212
	2	1	42.707	1.544	3.080
	2	1.5	39.917	1.297	2.769
	2.5	0.5	36.997	1.549	3.567
	2.5	1	32.131	1.267	3.358
	2.5	1.5	29.646	1.058	3.041

Supplementary Table 4 | Bill of components used in the flexible printed circuit board.

Components	Description	Footprint	Value and series number
C1, C2, C3, C4, C5, C6, C7, C8, C9, C10	Capacitor	0603	0.1 μF
C11	Capacitor	0603	0.22 μF
R1, R2, R3, R4, R5, R6, R7, R8, R9	Resistor	0603	10 kΩ
R10, R11, R12, R13	Resistor	0603	1 kΩ
R14, R15	Resistor	0603	300 Ω
R16	Resistor	0603	0 Ω
R17	Resistor	0805	0 Ω
PL1, PL2	LED	0603	
Q1, Q2	Bipolar Transistor	SOT23_3	PNP
U1	NFC	TSSOP-8	NT3H2111
U2	MCU	TQFP80/0.5	MSP430F5325
Y1	Crystal Oscillator	3215	32768Hz
J1	Header	HDR1X4	
FPCF	FPC/FFC connector		
MSC	MSC interface		
RF	Antenna interface		
KEY	Switch		
Psensor	Sensor interface		

FPC: flexible print circuit; FFC: flexible flat cable

Supplementary Table 5 | Areal capacitance comparison of various printed MSCs (Fig. S29b).

Materials	Fabrication method	Electrolyte	Areal capacitance per electrode (C/A, mF cm^{-2})	Ref.
Graphene	Inkjet printing	PSSH	0.7	49
Graphene	Spray coating	PVA/H_2SO_4	0.8	50
Graphene	3D printing	PVA/H_2SO_4	74.3	51
Graphene	Extrusion printing	PVA/H_2SO_4	19.8	27
GO/PANI	3D printing	PVA/H_3PO_4	153	29
CNT/PANI	3D printing	PMMA/PC/$LiClO_4$	44.1	52
CNT	3D printing	PVA/H_3PO_4	4.7	31
Ag@PPy@MnO_2	Screen printing	1 M Na_2SO_4	440	53
$Ti_3C_2T_x$ MXene	Extrusion printing	PVA/H_2SO_4	43	3
$Ti_3C_2T_x$ MXene	Pen writing	PVA/H_3PO_4	5	17
$Ti_3C_2T_x$ MXene	Inkjet printing	PVA/H_2SO_4	12	3
G-MXene	Spray coating	PVA/H_3PO_4	3.26	54
SA-MXene	Inkjet printing	PVA/H_2SO_4	108.1	55
$Ti_3C_2T_x$ MXene	Spray coating and laser cutting	PVA/H_2SO_4	23.4	56
$Ti_3C_2T_x$ MXene	Spray coating and laser cutting	PVA/H_2SO_4	27	57
MXene sediment	Screen printing	PVA/H_2SO_4	158	2
MXene-N	Screen printing	PVA/H_2SO_4	280.4	9
$Ti_3C_2T_x$ MXene	Extrusion printing	PVA/H_3PO_4	900	This work

PSSH: poly(4-styrenesulfonic acid); GO: graphene oxide; PANI: polyaniline; CNT: carbon nanotube; CNT-P: CNT-PANI; PMMA: (poly(methyl methacrylate)); PC: (propylene carbonate); PPy: polypyrrole; A-P-MnO_2: Ag@PPy@MnO_2; G-MXene: Graphene-MXene; SA: sodium ascorbate; MXene-N: nitrogen-doped MXene nanosheets

Supplementary Table 6 | Energy & power density comparison of various MSCs (Fig. 5f).

Materials	P (μW cm^{-2})	E (μWh cm^{-2})	Ref.
MXene	156.25	9.70	This work
	234.38	8.56	
	390.63	7.76	
	781.25	6.73	
	937.5	6.15	
	1406.25	4.84	
	1875	3.75	
GQD	7.5	0.074	58
rGO	9	0.014	59
PEDOT/Ag	138	0.041	60
	575	0.04	
	1480	0.034	
	2290	0.022	
	2880	0.019	
IJP-graphene	0.025	0.00139	61
	0.045	0.00125	
	0.1	0.00111	
	0.175	9.72E-04	
	0.3	8.33E-04	
sG-PEDOT	0.8	0.08889	60
	1.6	0.07111	
	3	0.06667	
	5.6	0.06222	
	12	0.05333	
	20.8	0.04622	
	40	0.04444	
GQD-MnO_2	7.5	0.154	58
S-graphene	0.2	0.02778	60
	0.40625	0.02257	
	0.7875	0.02187	
	1.35	0.01875	
	2.8125	0.01563	
	4	0.01111	
	5	0.00694	

Extrusion, MX	11.4	0.32	3
	22.5	0.31	
	44.1	0.30	
	75.1	0.21	
	115.4	0.16	
	157.7	0.11	
Stamped, MX	6.17	0.76	5
	14.06	0.74	
	30.31	0.73	
	64.78	0.71	
	135.36	0.68	
	326.44	0.63	
SP-MX	18.46	1.64	2
	37.11	1.62	
	75.57	1.57	
	156.98	1.51	
	334.99	1.42	
	544.12	1.36	
	778.33	1.32	

GOD: graphene quantum dot; rGO: reduced graphene oxide; PEDOT: Poly(3,4-ethylenedioxythiophene); IJP-graphene: inkjet-printed graphene; sG-PEDOT: spray-coated graphene/PEDOT; S-graphene: spray-coated graphene; Extrusion, MX: Extrusion-printed all-MXene MSC; Stamped, MX: stamped MXene MSC; SP-MX: Screen-printed MXene sediment ink

Supplementary Notes

Preparation of $Ti_3C_2T_x$ MXene aqueous inks.

The preparation of single-layer $Ti_3C_2T_x$ MXene aqueous inks was performed using a modified minimally intensive layer delamination (MILD) synthesis method, starting by selectively etching the Al layer of the Ti_3AlC_2 MAX phase. Briefly, under vigorous stirring at room temperature, 1 g of lithium fluoride (LiF, Sigma Aldrich, USA) was slowly added to 20 mL of 9 M hydrochloric acid (HCl, 37 wt%, Sigma Aldrich, USA) until the LiF was completely dissolved in HCl to form a clarifying solution. Then, 1 g of Ti_3AlC_2 MAX phase (<38 μm average particle size, Y-Carbon, Ukraine) was slowly added to the above mixture under vigorous stirring to prevent overheating, followed by a 24 h reaction at 35 °C with a stirring speed of 400 rpm. After etching, the mixture was transferred to a centrifuge tube and centrifuged at 3500 rpm for 5 min, then separated the supernatant from the sediment. Later, about 50 mL of fresh deionized water was added to the suspension and shaken vigorously for 1 min, followed by another round of centrifugation at 3500 rpm for 5 min. This washing process was repeated 4−5 times until the pH of the supernatant became ~6. To collect single-layer $Ti_3C_2T_x$ nanosheets, the etched MAX sediments were redispersed in deionized water and vigorously shaken by a vortex machine for 30 min. Afterward, the mixture was centrifuged at 3500 rpm for 1 h and discarded sediment containing the unetched MAX phase and unexfoliated $Ti_3C_2T_x$. The fully delaminated MXene nanosheets were collected in the supernatant and further centrifuged at 7000 rpm for 1 h and discarded the supernatant, which contains relatively small flakes. As a control of conventional inks, the MXene ink without flake diameter screening was obtained by setting two centrifugation rates at 1500 rpm and 12000 rpm. Finally, the obtained $Ti_3C_2T_x$ MXene aqueous ink was obtained by redispersing the sediment in deionized water and

sonicating it for 15 min in an ice bath under argon bubbling. The concentration of $Ti_3C_2T_x$ ink was controlled at ~60 mg mL^{-1} by the addition of water to achieve appropriate rheological properties for direct extrusion printing.

Scanning electron microscopy.

SEM images of the printed lines and patterns were acquired using a Zeiss Ultra Plus (Carl Zeiss, Germany). The cross-section SEM image was obtained by tearing the MXene film with a tweezer. The printing spatial uniformity is characterized by the width variation, which was obtained from the width statistics of as-printed MXene lines along the axial direction.

Transmission electron microscopy.

The morphology of delaminated MXene nanosheets was obtained using transmission electron microscopy (TEM, FEI Titan, USA) with an operating voltage of 300 kV. TEM samples were prepared by drop-casting the diluted MXene aqueous inks onto the ultra-thin holey carbon copper grids (400 mesh - Agar). The size histogram of MXene nanosheets was obtained from the statistical data of ~270 MXene flakes in TEM images. Energy-dispersive X-ray spectroscopy (EDX) was measured through the EDAX detector during the TEM measurement.

X-ray diffraction.

X-ray diffraction (XRD) patterns were acquired through a Bruker D8 Advance Diffractometer (Germany) with a working voltage of 40 kV. The scanning range was set from 5° to 65° with a step size of $2\theta = 0.02°$.

Dynamic light scattering measurement.

DLS measurements were performed at room temperature by pipetting 1 mL of MXene dispersion into a polystyrene cuvette (Zetasizer Nano ZS90, Malvern, UK).

Atomic force microscopy.

Atomic force microscopy (AFM) images were taken in peak force tapping mode under ambient conditions by a Dimension Icon AFM (Bruker AXS, Germany). Height profiles of the MXene nanosheets were processed with Nanoscope analysis and Gwyddion software. The AFM measurements of MXene nanosheets were performed on the silicon wafer substrate.

White-light interference measurement.

Height profiles of the as-printed MXene lines and films were acquired using an optical profilometer (WLI, Wyko NT9100, Veeco, USA), which were directly measured on the plasma-treated PET without other modification. The height and surface roughness (Ra) of the MXene films was accessed by the Vision software.

Raman measurement.

Raman spectra of as-printed MXene films were obtained using a confocal Raman microscope system (LabRAM HR Evolution, HORIBA Jobin Yvon, France). The excitation wavelength was 633 nm, and the laser power was ~170 μW.

Rheological characterization.

The rheological behavior of $Ti_3C_2T_x$ MXene inks was measured with an Anton Paar MCR 301 rheometer using a PP25 (parallel plate geometry, diameter of 25 mm, gap of 0.5 mm). All the loaded samples were equilibrated for 1 minute at room temperature. Viscosities variation was recorded as

a function of shear rates via steady-state continuous shear experiments with a sweep of shear rate (0.01-1000 s^{-1}) at a frequency of 1Hz. For the simulation of the extrusion printing process, alternate low (0.1 s^{-1}, 45 s) and high (100 s^{-1}, 45s and 90 s) shear rates were applied to the MXene ink. To evaluate the rheological properties, the storage modulus (G′) and loss modulus (G″) of MXene aqueous ink were measured as functions of shear stress (0.1-200 Pa). The storage modulus (G′) and loss modulus (G″) were also measured as functions of the frequency with 1% strain amplitude to calculate the frequency dependence of the G′/G″ ratio.

Sheet resistance and electrical measurement.

The sheet resistance (R_s) of the as-printed MXene films was measured using the ST2263 double testing digital four-point probe tester (Suzhou Jingge Electronic Co., Ltd, Suzhou, China) at ambient temperature. Each test was performed at least three times to calculate the average value and standard deviation. Electrical conductivity (σ) of printed MXene film can be calculated from the measured R_s and thickness (t):

$$\sigma = \frac{1}{R_s \cdot t} \tag{1}$$

The electrical conductivity under different drying conditions was determined by measuring the corresponding sheet resistance, R_s. The measurement was carried out after the initial printing of MXene film at ambient conditions (25 °C, 40% RH) for 10 min and further drying in low humidity conditions (<10% RH) for another 4 hours, respectively. The continuity test between printed MXene lines was performed on a Keithley 4200A-SCS Parameter Analyzer with a probe station.

Mechanical flexibility measurement.

The bending test was applied for the measurement of the mechanical flexibility of the as-printed MXene lines (2 mm × 70 mm on PI and PDMS substrates). Firstly, two pieces of Ag wire were fixed

onto both ends of the MXene lines using MXene clay as a conductive adhesive. Then, the connecting point was covered and secured by the scotch tape to ensure its robustness during the bending test. The resistance variations of the MXene line under different bending degrees were recorded by a digital multimeter (Keithley DMM 7510). One cycle is defined as bending the flat MXene line to 180° and then releasing it to the flat state. For the cycling test, the MXene line was bent for 1000 cycles, and the resistance was recorded accordingly.

Preparation of high-precision conductive pattern.

The extrusion printing process for the fabrication of high-precision conductive patterns was performed using a programmable three-axis pneumatic robotic deposition system (SM300ΩX-3A-SS, MUSASHI) with PC control image recognition (IMAGE MASTER 350PC Smart). Computer painting software (MuCAD, MUSASHI) allows the adjustment of extrusion printing parameters to produce different patterns according to requirements. During the printing process, the cylindrical extrusion nozzle can move in the three-axis direction at a preset speed following a pre-programmed printing program. The MXene ink is extruded through the fine cylindrical nozzle (inner diameter of 110 μm, standard size 32G) under a set pressure (10 kPa) and moving speed (10–30 mm s^{-1}) and deposited directly on specific substrates (e.g., PI, PET, and PDMS) under ambient conditions. Beforehand, the polymer and glass substrates were subjected to air plasma treatment with a plasma cleaner (PDC-002, Harrick Plasma) for 5 min to improve the wettability. The ferrite substrate and plant leaves are used directly without treatment. The distance between the nozzle and the substrate was maintained at 0.1 mm to ensure proper adhesion. By adjusting the printing number $<N>$, the desired MXene film thickness can be obtained. The entire printing process is carried out at room

temperature without additional heating, and no high-temperature annealing or other post-processing procedures are required after the printing is completed.

Fabrication of NFC and RFID electronics.

According to the preset program, the functional conductive patterns (NFC antennas and dipole antennas) were first fabricated by the extrusion printing of MXene ink (<*N*> = 15). For the fabrication of the NFC tags, 300 μm PDMS was selected as the substrate, and PU adhesive tape was used as the insulating film. After mounting the NFC chip (NTAG 215 or FM11RF08) and the indicative LED, the NFC tag was encapsulated with a 200 μm PDMS film. For the fabrication of the RFID temperature tags, the substrate was 300 μm PDMS or PET. The dipole antenna dimension was fixed at 15 mm × 86 mm, predesigned by the simulation software CST Microwave Studio. After mounting the RFID chip (NMV2D CAB0), a 200 μm PDMS film was used to complete the tag encapsulation. During the fabrication process, the conductive joints were all yielded by using conductive MXene pastes without any other conductive adhesives.

Mechanical and electrodynamic simulations for NFC and RFID electronics.

FEA analysis was performed to simulate the strain distribution of the flexible MXene NFC tag under bending *via* the FEA commercial software COMSOL Multiphysics (version 5.5, COMSOL Inc.). The effects of geometrical parameters on the performance of MXene NFC tags were evaluated by the commercial full-wave electromagnetic simulation software Ansys HFSS (Canonsburg, PA, USA). The Ansys HFSS was also used to simulate the current distribution of the MXene NFC antenna at 13.56 MHz and the relationship between resistance, Q factor, and printed thickness. The MXene dipole antenna's geometrical parameters and current distribution at the frequency of 920 MHz were determined by commercial full-wave electromagnetic simulation software CST

Microwave Studio (Dassault Systems).

RFID antenna measurement.

The reflection coefficient was measured using an Agilent 8722ES vector network analyzer to quantify the return loss of the MXene dipole antenna from 0.7 to 1.2 GHz. A SubMiniature version A (SMA) connector with coaxial cables was used to feed the MXene dipole antenna. To evaluate the flexibility and stability, the MXene dipole antenna was measured after 45° bending of 0, 200, and 500 times, respectively. The radiation pattern measurement was carried out at a frequency of 920 MHz in an anechoic chamber room. The MXene dipole antenna was mounted on a rotary table and measured with a step size of 1°. A double-ridged horn antenna was used as the receiving antenna.

MXene RFID tags for surface temperature monitoring.

For monitoring local leaf temperature, RFID Tags 1 to 3 are manufactured on soft PDMS, allowing them to adhere tightly to the surface of the plant leaves. Tag 4 is manufactured on flexible PET, allowing it to be inserted into the soil for temperature monitoring. During the test, an electric hair dryer was used to generate hot air to simulate the local temperature change. The higher temperature of tag 4 is probably due to the rapid heating of the electric hairdryer after starting. For human body surface temperature monitoring, RFID tags were used as the wearable sensors on the human wristband (inset), forehead, and chest. Since the measurement results directly reflect the ambient temperature of the human body surface, the temperature measured in different body parts is accordingly distinct and tends to be lower than the normal human body temperature.

Fabrication of the all-MXene-printed flexible integrated NFC-enabled sensing system.

For the preparation of wireless, energy, and sensing modules, three functional conductive patterns

were integrated and manufactured on PDMS by the direct printing of MXene ink according to a predefined computer program. The overall dimensions of the functional MXene patterns are 30 mm × 110 mm. The external control FPCB was mounted onto the functionalized MXene patterns to connect three available modules. The contacts were ensured using MXene clay as the conductive adhesive and secured with transparent PU tape. Finally, the FPCB and the contacts between the FPCB and the functional modules were further fixed by coating with PDMS (Sylgard 184, Dow Corning Corp., 10:1 weight ratio).

Design of the FPCB and Mobile Application.

The NFC chip (NT3H2111, NXP Semiconductor, Netherlands) and the MCU module (MSP430F5325, Texas Instruments, USA) are the two core electronic components of the custom FPCB. The three contacts of the FPCB are used to connect the MXene printed wireless, energy, and sensing modules, respectively. The electromagnetic induction of the antennas between the NFC reader and device can wirelessly transmit power to the NFC chip, which is rectified by the power management module into a stable voltage output. Referring to the datasheet, the NFC chip's maximum output power can reach15 mW, and its no-load voltage output is about 3.3 V. The ultra-low-power MCU has a 64 KB nonvolatile memory and a 12-bit A/D converter, with a power supply voltage range of 1.8 to 3.6 V, which matches well with the NFC chip' voltage output. The operating current in ultralow-power mode is about 100 $\mu A\ MHz^{-1}$. The internal A/D converter of the MCU is responsible for converting the current signals from temperature and humidity sensors into digital signals for further transmission to the EEPROM of the NFC chip, which will eventually be wirelessly sent to the smartphone according to the ISO/IEC 14443 standard. In this work, a smartphone (Mi 6) was used as the NFC reader for wireless power and data transmission. The actual

operating voltage output of the NFC chip was about 3 V, enough for most electronic devices. In the experiment, for improving the antenna coupling efficiency, the smartphone and the device antenna were as close as possible to enhance the field strength between the NFC antennas. The custom Android mobile app, called MXene NFC sensing system, was built using Android Studio and contained three primary interfaces: real-time T/H data, recorded historical curves, and system settings. According to the fitting sensor equations, the digital signal data transmitted to the smartphone was further converted into the specific T/H values in the mobile application. In practice, when the user launched the mobile app and successfully paired the smartphone with the device, the real-time T/H values would be displayed on the smartphone interface. All data can be shared via social media.

Fabrication of on-chip MXene MSCs.

The MSC unit and module were fabricated based on the as-printed $Ti_3C_2T_x$ MXene interdigitated patterns ($<N> = 15$), respectively. No additional current collectors, conductive agents, or polymer binders are required during the entire manufacturing process, as $Ti_3C_2T_x$ possesses excellent metallic conductivity, allowing it to serve as both the active material and current collector of MSCs. For the preparation of phosphoric acid (H_3PO_4)-poly(vinyl alcohol, PVA) gel electrolyte, 1 g PVA (M_w = 89,000–98,000, Sigma-Aldrich) was weighed and dissolved in 10 mL deionized H_2O under constant stirring at 80 °C for 4 h to obtain a transparent gel solution. Then, 1 g (0.6 mL) of concentrated phosphoric acid (Alfa Aesar, 85%) was added to 10 wt% PVA gel and stirred for 1 h to obtain PVA/H_3PO_4 gel electrolyte. The final solution was cooled to room temperature and dropped onto the interdigital pattern area of the printed MXene MSCs to completely wet the electrode and then dried under ambient conditions for electrolyte solidification. Two conductive tapes were attached at

the end of the MSC unit or module and glued with MXene clay to facilitate the electrochemical measurements.

Electrochemical characterization.

The electrochemical performance of the MXene MSCs was investigated by CV curves, GCD profiles, and electrochemical impedance spectra (EIS) measurements, all of which were performed on a CHI 760E electrochemical workstation (Chenhua Instruments Co., Shanghai, China). For one unit of the MSC, the CV scan rates and GCD current densities in a voltage window of 0–0.6 V were set to 1 to 200 mV s^{-1} and 0.8 to 6.3 mA cm^{-2}, respectively. For the MSC module, the CV scan rates and GCD current densities in a voltage window of 3V were set to 50 to 500 mV s^{-1} and 0.8 to 3.6 mA, respectively. The cycling performance of the MSC module was evaluated at 7.2 mA for 3000 cycles. EIS measurements were carried out at the open-circuit voltage ranging from 10 kHz to 100 mHz.

The capacitance value C (mF) based on the CV curve was calculated according to the following equation:

$$C = \frac{1}{v\Delta V}\int_{V_1}^{V_2} i(V)\mathrm{d}V \tag{2}$$

where V_2 and V_1 are the bounds of the voltammetric curve, ΔV is the potential window ($\Delta V = V_2 - V_1$), v is the scan rate (mV s^{-1}), $i(V)$ is the voltammetric discharge current (mA) measured during CV testing, and $\mathrm{d}V$ is infinitesimal changes in potential.

The capacitance values C (mF cm^{-2}) based on the GCD curve was calculated by the following equation:

$$C = \frac{I \times \Delta t}{\Delta V} \tag{3}$$

where I is the discharge current (mA), Δt is the discharge time (s), and ΔV is the discharge potential

window (V).

The areal capacitance C/A (mF cm^{-2}) pre electrode was calculated by the following equation:

$$C/\mathrm{A} = \frac{4C}{A} \tag{4}$$

where A (cm^2) is the total geometric area of MSCs, involving the finger electrodes and interspace regions.

For the Ragone plot, the device areal energy density (E, μWh cm^{-2}) and power density (P, mWh cm^{-2}) were calculated using the following equation:

$$E = \frac{C \times (\Delta V)^2}{\mathrm{A} \times 2 \times 3.6} \tag{5}$$

$$P = \frac{E \times 3.6}{\Delta t} \tag{6}$$

where ΔV is the discharge potential window (V), and Δt is the discharge time (s).

The device coulombic efficiency (CE) of the charge-discharge cycling is calculated by the following equation:

$$CE = \frac{t_{discharge}}{t_{charge}} \times 100\% \tag{7}$$

where $t_{\mathrm{discharge}}$ (s) and t_{charge} (s) are the time spent for discharging or charging between the maximum voltage and 0 V, respectively.

Fabrication of all-MXene-printed temperature and humidity sensing module.

For the fabrication of the MXene temperature sensor, a layer of PDMS was used to cover the MXene ink printed conductive curved channel ($\langle N \rangle = 1$) as an encapsulation layer to avoid the humidity effect. For the fabrication of the MXene humidity sensor, 50 ml MXene aqueous solution with a concentration of 0.5 mg mL^{-1} was drop-casting on the surface of MXene printed interdigital electrode ($\langle N \rangle = 2$) and then placed at room temperature for water evaporation to obtain the uniform MXene humidity sensing thin film.

Characterization of the MXene temperature and humidity sensors.

The temperature sensor characterization was performed on a hot plate in the range of 20–55 °C. The sensor resistance changes were measured using a digital multimeter (Keithley DMM 7510) and calibrated with the reading of a thermometer (Hti HT 9815). The temperature monitoring of palm or hot wind was measured by observing the normalized electrical resistance change

$$\frac{\Delta R}{R_0} \times 100\% \tag{8}$$

where $\Delta R = R - R_0$, and R and R_0 are the measured and initial resistance before measurement, respectively. The temperature sensitivity is defined as

$$\frac{\Delta R}{R_0} \times \frac{1}{\Delta T} \times 100\% \tag{9}$$

where ΔT is the temperature variation. The sensitivity of the MXene temperature sensor in the range of 20–55 °C was normalized with the resistance at 20 °C. The humidity sensing measurement was carried out in a homemade testing system. The MXene humidity sensor was first placed in a hermetic metallic testing chamber with a three-way air hole as the inlet and outlet for N_2 gas. Dry N_2 from the gas cylinder was mixed with wet N_2 that flowed through the DI water and then entered the chamber. Through adjusting the rate ratio of these two N_2 flows by the gas flow rate controller (CDLaiFeng LF-1A), the relative humidity (calibrated with a commercial hygrometer, TASI 621) in the chamber can be manually controlled as required. A digital multimeter (Keithley DMM 7510) was used to measure and record the electrical resistance response of the MXene humidity sensor. The response time of the humidity sensor is defined as the time to reach 90 % of the final steady resistance value.

Supplementary References

1. Zhang, C. F. et al. Transparent, flexible, and conductive 2D titanium carbide (MXene) films with high volumetric capacitance. *Adv. Mater.* **29**, 1702678 (2017).
2. Abdolhosseinzadeh, S., Schneider, R., Verma, A., Heier, J., Nuesch, F. & Zhang, C. J. Turning trash into treasure: additive free MXene sediment inks for screen-printed micro-supercapacitors. *Adv. Mater.* **32**, e2000716 (2020).
3. Zhang, C. J. et al. Additive-free MXene inks and direct printing of micro-supercapacitors. *Nat. Commun.* **10**, 1795 (2019).
4. Maleski, K., Ren, C. E., Zhao, M. Q., Anasori, B. & Gogotsi, Y. Size-dependent physical and electrochemical properties of two-dimensional MXene flakes. *ACS Appl. Mater. Interfaces* **10**, 24491-24498 (2018).
5. Zhang, C. et al. Stamping of flexible, coplanar micro-supercapacitors using MXene inks. *Adv. Funct. Mater.* **28**, 1705506 (2018).
6. Uzun, S. et al. Additive-free aqueous MXene inks for thermal inkjet printing on textiles. *Small* **17**, 2006376 (2020).
7. Piatti, E. et al. Charge transport mechanisms in inkjet-printed thin-film transistors based on two-dimensional materials. *Nat. Electron.* **4**, 893-905 (2021).
8. Lan, L., Le, X., Dong, H., Xie, J., Ying, Y. & Ping, J. One-step and large-scale fabrication of flexible and wearable humidity sensor based on laser-induced graphene for real-time tracking of plant transpiration at bio-interface. *Biosens. Bioelectron.* **165**, 112360 (2020).
9. Yu, L., Fan, Z., Shao, Y., Tian, Z., Sun, J. & Liu, Z. Versatile N-doped MXene ink for printed electrochemical energy storage application. *Adv. Energy Mater.* **9**, 1901839 (2019).

10. Shen, K., Li, B. & Yang, S. 3D printing dendrite-free lithium anodes based on the nucleated MXene arrays. *Energy Stor. Mater.* **24**, 670-675 (2020).

11. Orangi, J., Hamade, F., Davis, V. A. & Beidaghi, M. 3D printing of additive-free 2D $Ti_3C_2T_x$ (MXene) ink for fabrication of micro-supercapacitors with ultra-high energy densities. *ACS Nano* **14**, 640-650 (2020).

12. Fan, Z. et al. 3D printing of porous nitrogen-doped Ti_3C_2 MXene scaffolds for high-performance sodium-ion hybrid capacitors. *ACS Nano* **14**, 867-876 (2020).

13. Yang, W. et al. 3D printing of freestanding MXene architectures for current-collector-free supercapacitors. *Adv. Mater.* **31**, e1902725 (2019).

14. Fan, Z. et al. 3D-printed Zn-ion hybrid capacitor enabled by universal divalent cation-gelated additive-free Ti_3C_2 MXene ink. *ACS Nano* **15**, 3098-3107 (2021).

15. Huang, X., Huang, J., Yang, D. & Wu, P. A multi-scale structural engineering strategy for high-performance MXene hydrogel supercapacitor electrode. *Adv. Sci.* **8**, e2101664 (2021).

16. Jambhulkar, S. et al. Aligned $Ti_3C_2T_x$ MXene for 3D micropatterning via additive manufacturing. *ACS Nano* **15**, 12057-12068 (2021).

17. Quain, E. et al. Direct writing of additive-free MXene-in-water ink for electronics and energy storage. *Adv. Mater. Technol.* **4**, 1800256 (2019).

18. Tang, X. et al. Engineering aggregation-resistant MXene nanosheets as highly conductive and stable inks for all-printed electronics. *Adv. Funct. Mater.* **31**, 2010897 (2021).

19. Jiang, X. T. et al. Inkjet-printed MXene micro-scale devices for integrated broadband ultrafast photonics. *npj 2D Mater. Appl.* **3**, 34 (2019).

20. Vural, M. et al. Inkjet printing of self-assembled 2D titanium carbide and protein electrodes

for stimuli-responsive electromagnetic shielding. *Adv. Funct. Mater.* **28**, 1801972 (2018).

21. Ma, J. X. et al. Aqueous MXene/PH1000 hybrid inks for inkjet-printing micro-supercapacitors with unprecedented volumetric capacitance and modular self-powered microelectronics. *Adv. Energy Mater.* **11**, 2100746 (2021).

22. Xu, B. et al. Ultrathin MXene-micropattern-based field-effect transistor for probing neural activity. *Adv. Mater.* **28**, 3333-3339 (2016).

23. Jiang, Q. et al. On-chip MXene microsupercapacitors for AC-line filtering applications. *Adv. Energy Mater.* **9**, 1901061 (2019).

24. Driscoll, N. et al. Two-dimensional Ti_3C_2 MXene for high-resolution neural interfaces. *ACS Nano* **12**, 10419-10429 (2018).

25. Li, H. P., Li, X. R., Liang, J. J. & Chen, Y. S. Hydrous RuO_2-decorated MXene coordinating with silver nanowire inks enabling fully printed micro-supercapacitors with extraordinary volumetric performance. *Adv. Energy Mater.* **9**, 1803987 (2019).

26. Secor, E. B., Gao, T. Z., Dos Santos, M. H., Wallace, S. G., Putz, K. W. & Hersam, M. C. Combustion-assisted photonic annealing of printable graphene inks via exothermic binders. *ACS Appl. Mater. Interfaces* **9**, 29418-29423 (2017).

27. Sun, G., An, J., Chua, C. K., Pang, H., Zhang, J. & Chen, P. Layer-by-layer printing of laminated graphene-based interdigitated microelectrodes for flexible planar micro-supercapacitors. *Electrochem. Commun.* **51**, 33-36 (2015).

28. Tang, X. et al. Generalized 3D printing of graphene-based mixed-dimensional hybrid aerogels. *ACS Nano* **12**, 3502-3511 (2018).

29. Liu, Y. Q. et al. Development of graphene oxide/polyaniline inks for high performance

flexible microsupercapacitors via extrusion printing. *Adv. Funct. Mater.* **28**, 1706592 (2018).

30. Pidcock, G. C. & in het Panhuis, M. Extrusion printing of flexible electrically conducting carbon nanotube networks. *Adv. Funct. Mater.* **22**, 4790-4800 (2012).

31. Yu, W., Zhou, H., Li, B. Q. & Ding, S. 3D printing of carbon nanotubes-based microsupercapacitors. *ACS Appl. Mater. Interfaces* **9**, 4597-4604 (2017).

32. Owens, C. E. et al. Substrate-versatile direct-write printing of carbon nanotube-based flexible conductors, circuits, and sensors. *Adv. Funct. Mater.* **31**, 2100245 (2021).

33. Zhu, Z. et al. 3D printed functional and biological materials on moving freeform surfaces. *Adv. Mater.* **30**, e1707495 (2018).

34. Lopes, P. A., Santos, B. C., de Almeida, A. T. & Tavakoli, M. Reversible polymer-gel transition for ultra-stretchable chip-integrated circuits through self-soldering and self-coating and self-healing. *Nat. Commun.* **12**, 4666 (2021).

35. Valentine, A. D. Hybrid 3D printing of soft electronics. *Adv. Mater.* **29**, 1703817 (2017).

36. Kim, F. et al. Direct ink writing of three-dimensional thermoelectric microarchitectures. *Nat. Electron.* **4**, 579-587 (2021).

37. Yuk, H. et al. 3D printing of conducting polymers. *Nat. Commun.* **11**, 1604 (2020).

38. Chao, Y. et al. One-pot hydrothermal synthesis of solution-processable MoS_2/PEDOT:PSS composites for high-performance supercapacitors. *ACS Appl. Mater. Interfaces* **13**, 7285-7296 (2021).

39. Hong, H., Hu, J. & Yan, X. UV curable conductive ink for the fabrication of textile-based conductive circuits and wearable UHF RFID tags. *ACS Appl. Mater. Interfaces* **11**, 27318-

27326 (2019).

40. Matsuhisa, N. et al. Printable elastic conductors with a high conductivity for electronic textile applications. *Nat. Commun.* **6**, 7461 (2015).

41. Li, Z. et al. Rational design of a printable, highly conductive silicone-based electrically conductive adhesive for stretchable radio-frequency antennas. *Adv. Funct. Mater.* **25**, 464-470 (2015).

42. Feng, S., Tian, Z., Wang, J., Cao, S. & Kong, D. Laser sintering of Zn microparticles and its application in printable biodegradable electronics. *Adv. Electron. Mater.* **5**, 1800693 (2019).

43. Lee, Y. K., Kim, J., Kim, Y., Kwak, J. W., Yoon, Y. & Rogers, J. A. Room temperature electrochemical sintering of Zn microparticles and its use in printable conducting inks for bioresorbable electronics. *Adv. Mater.* **29**, 1702665 (2017).

44. Tang, L. et al. Printable metal-polymer conductors for highly stretchable bio-devices. *iScience* **4**, 302-311 (2018).

45. Veerapandian, S. et al. Hydrogen-doped viscoplastic liquid metal microparticles for stretchable printed metal lines. *Nat. Mater.* **20**, 533-540 (2021).

46. Lopes, P. A. et al. Bi-phasic Ag-In-Ga-embedded elastomer inks for digitally printed, ultra-stretchable, multi-layer electronics. *ACS Appl. Mater. Interfaces* **13**, 14552-14561 (2021).

47. Li, Y., Feng, S., Cao, S., Zhang, J. & Kong, D. Printable liquid metal microparticle ink for ultrastretchable electronics. *ACS Appl. Mater. Interfaces* **12**, 50852-50859 (2020).

48. Liu, S., Shah, D. S. & Kramer-Bottiglio, R. Highly stretchable multilayer electronic circuits using biphasic gallium-indium. *Nat. Mater.* **20**, 851-858 (2021).

49. Li, J. T. et al. Scalable fabrication and integration of graphene microsupercapacitors through full inkjet printing. *ACS Nano* **11**, 8249-8256 (2017).

50. Liu, Z., Wu, Z. S., Yang, S., Dong, R., Feng, X. & Mullen, K. Ultraflexible in-plane micro-supercapacitors by direct printing of solution-processable electrochemically exfoliated graphene. *Adv. Mater.* **28**, 2217-2222 (2016).

51. Li, W. et al. Printing assembly and structural regulation of graphene towards three-dimensional flexible micro-supercapacitors. *J. Mater. Chem. A* **5**, 16281-16288 (2017).

52. Li, L., Lou, Z., Han, W., Chen, D., Jiang, K. & Shen, G. Highly stretchable micro-supercapacitor arrays with hybrid MWCNT/PANI electrodes. *Adv. Mater. Technol.* **2**, 1600282 (2017).

53. Liu, L., Tian, Q., Yao, W., Li, M., Li, Y. & Wu, W. All-printed ultraflexible and stretchable asymmetric in-plane solid-state supercapacitors (ASCs) for wearable electronics. *J. Power Sources* **397**, 59-67 (2018).

54. Li, H. et al. Flexible all-solid-state supercapacitors with high volumetric capacitances boosted by solution processable MXene and electrochemically exfoliated graphene. *Adv. Energy Mater.* **7**, 1601847 (2017).

55. Wu, C.-W., Unnikrishnan, B., Chen, I.-W. P., Harroun, S. G., Chang, H.-T. & Huang, C.-C. Excellent oxidation resistive MXene aqueous ink for micro-supercapacitor application. *Energy Stor. Mater.* **25**, 563-571 (2020).

56. Huang, H. C. et al. Massively manufactured paper-based all-solid-state flexible micro-supercapacitors with sprayable MXene conductive inks. *J. Power Sources* **415**, 1-7 (2019).

57. Peng, Y.-Y. et al. All-MXene (2D titanium carbide) solid-state microsupercapacitors for on-

chip energy storage. *Energy Environ. Sci.* **9**, 2847-2854 (2016).

58. Liu, W.-W., Feng, Y.-Q., Yan, X.-B., Chen, J.-T. & Xue, Q.-J. Superior micro-supercapacitors based on graphene quantum dots. *Adv. Funct. Mater.* **23**, 4111-4122 (2013).

59. Yoo, J. J. et al. Ultrathin planar graphene supercapacitors. *Nano Lett.* **11**, 1423-1427 (2011).

60. Kurra, N., Ahmed, B., Gogotsi, Y. & Alshareef, H. N. MXene-on-paper coplanar microsupercapacitors. *Adv. Energy Mater.* **6**, 1601372 (2016).

61. Sollami Delekta, S., Smith, A. D., Li, J. & Ostling, M. Inkjet printed highly transparent and flexible graphene micro-supercapacitors. *Nanoscale* **9**, 6998-7005 (2017).
