# Supplementary material for: Room-temperature high-precision printing of flexible wireless electronics based on MXene inks
Source: Nat Commun. 2022 Jun 9;13:3223. doi: 10.1038/s41467-022-30648-2 (PMC9184614; doi:10.1038/s41467-022-30648-2)
Supplement: Supplementary file 3 — Description of Additional Supplementary Files [file 41467_2022_30648_MOESM3_ESM.docx]

**Description of Additional Supplementary Files**

**File Name: Supplementary Movie 1**

**Description:** Direct extrusion printing of MXene circuits and conformal printing on irregular plant leaves.

**File Name: Supplementary Movie 2**

**Description:** Demonstration of the excellent flexibility of MXene NFC tag.

**File Name: Supplementary Movie 3**

**Description:** Wireless energy harvesting to light up hundreds of parallel LEDs.

**File Name: Supplementary Movie 4**

**Description:** Applications of MXene NFC tags: access card for standard electronic door locks and identification label for plant information.

**File Name: Supplementary Movie 5**

**Description:** Wireless RFID tags used for monitoring temperature variation in the local microenvironment of plants.

**File Name: Supplementary Movie 6**

**Description:** Operation demonstration of the all-MXene-printed integrated sensing system for temperature and humidity monitoring.
